# Population‐level impact of expanding PrEP coverage by offering long‐acting injectable PrEP to MSM in three high‐resource settings: a model comparison analysis

**DOI:** 10.1002/jia2.26109

**Published:** 2023-07-13

**Authors:** Sarah E. Stansfield, Jesse Heitner, Kate M. Mitchell, Carla M. Doyle, Rachael M. Milwid, Mia Moore, Deborah J. Donnell, Brett Hanscom, Yiqing Xia, Mathieu Maheu‐Giroux, David van de Vijver, Haoyi Wang, Ruanne Barnabas, Marie‐Claude Boily, Dobromir T. Dimitrov

**Affiliations:** ^1^ Fred Hutchinson Cancer Center Seattle Washington USA; ^2^ Massachusetts General Hospital Boston Massachusetts USA; ^3^ HIV Prevention Trials Network Modelling Centre Imperial College London London UK; ^4^ Department of Nursing and Community Health Glasgow Caledonian University London London UK; ^5^ MRC Centre for Global Infectious Disease Analysis, School of Public Health Imperial College London London UK; ^6^ Department of Epidemiology and Biostatistics, School of Population and Global Health McGill University Montréal Québec Canada; ^7^ University of Washington Seattle Washington USA; ^8^ Viroscience Department Erasmus Medical Centre Rotterdam the Netherlands; ^9^ Department of Work and Social Psychology Maastricht University Maastricht the Netherlands

**Keywords:** HIV prevention, men who have sex with men, modelling, PrEP, North America, Europe

## Abstract

**Introduction:**

Long‐acting injectable cabotegravir (CAB‐LA) demonstrated superiority to daily tenofovir disoproxil fumarate/emtricitabine (TDF/FTC) for HIV pre‐exposure prophylaxis (PrEP) in the HPTN 083/084 trials. We compared the potential impact of expanding PrEP coverage by offering CAB‐LA to men who have sex with men (MSM) in Atlanta (US), Montreal (Canada) and the Netherlands, settings with different HIV epidemics.

**Methods:**

Three risk‐stratified HIV transmission models were independently parameterized and calibrated to local data. In Atlanta, Montreal and the Netherlands, the models, respectively, estimated mean TDF/FTC coverage starting at 29%, 7% and 4% in 2022, and projected HIV incidence per 100 person‐years (PY), respectively, decreasing from 2.06 to 1.62, 0.08 to 0.03 and 0.07 to 0.001 by 2042. Expansion of PrEP coverage was simulated by recruiting new CAB‐LA users and by switching different proportions of TDF/FTC users to CAB‐LA. Population effectiveness and efficiency of PrEP expansions were evaluated over 20 years in comparison to baseline scenarios with TDF/FTC only.

**Results:**

Increasing PrEP coverage by 11 percentage points (pp) from 29% to 40% by 2032 was expected to avert a median 36% of new HIV acquisitions in Atlanta. Substantially larger increases (by 33 or 26 pp) in PrEP coverage (to 40% or 30%) were needed to achieve comparable reductions in Montreal and the Netherlands, respectively. A median 17 additional PYs on PrEP were needed to prevent one acquisition in Atlanta with 40% PrEP coverage, compared to 1000+ in Montreal and 4000+ in the Netherlands. Reaching 50% PrEP coverage by 2032 by recruiting CAB‐LA users among PrEP‐eligible MSM could avert >45% of new HIV acquisitions in all settings. Achieving targeted coverage 5 years earlier increased the impact by 5–10 pp. In the Atlanta model, PrEP expansions achieving 40% and 50% coverage reduced differences in PrEP access between PrEP‐indicated White and Black MSM from 23 to 9 pp and 4 pp, respectively.

**Conclusions:**

Achieving high PrEP coverage by offering CAB‐LA can impact the HIV epidemic substantially if rolled out without delays. These PrEP expansions may be efficient in settings with high HIV incidence (like Atlanta) but not in settings with low HIV incidence (like Montreal and the Netherlands).

## 1 INTRODUCTION

Two clinical studies (HPTN 083 and HPTN 084) have shown that long‐acting injectable cabotegravir (CAB‐LA) pre‐exposure prophylaxis (PrEP) given every 8 weeks is highly efficacious at preventing HIV among cisgender men who have sex with men (MSM) and transgender and cisgender women in several countries [1, 2]. This new HIV prevention option may help increase PrEP coverage as it may be more appealing to people at risk who do not want or are unable to use daily oral tenofovir disoproxil fumarate/emtricitabine (TDF/FTC) PrEP. In trial settings, participants have found CAB‐LA to be acceptable, with many participants interested in continuing injections [3, 4, 5]. Currently, CAB‐LA is approved for use in the United States, though not yet in Canada or the Netherlands [6], and the World Health Organization (WHO) has recently recommended CAB‐LA as an additional prevention choice for people at substantial risk of HIV acquisition [7, 8].

Many factors need to be considered to determine how to optimize the use of CAB‐LA after its approval by regulatory authorities. Given that CAB‐LA will be offered alongside other HIV prevention options, including daily TDF/FTC [9, 10], it is important to determine who will have access to CAB‐LA based on local eligibility criteria [11, 12, 13] to predict what proportion of current TDF/FTC users may decide to switch to CAB‐LA [14] and how many additional PrEP users may need to be recruited to more substantially impact HIV incidence after CAB‐LA becomes available. It is critical that the introduction of CAB‐LA does not result in decreases in PrEP coverage by creating additional barriers to service access or by negatively affecting integration with existing HIV prevention options [15].

Aiming to address questions about the impact of PrEP scale‐up by offering CAB‐LA alongside TDF/FTC, the HPTN Modelling Centre (https://hptnmodelling.org/) and the HIV Modelling Consortium collaborated to conduct a model‐based comparison of the expected population‐level impact of introducing CAB‐LA in different geographic areas with different HIV incidence and current PrEP coverage levels and in selected risk populations. The goal of this project is to use existing, well‐calibrated, transmission‐dynamic models of HIV to predict and compare the population‐level effectiveness and efficiency of expanding overall PrEP coverage by adding CAB‐LA to the HIV prevention portfolio under prioritized and access‐to‐all strategies. The results of this study were used to inform current WHO recommendations [16] and could provide the basis for regional, national and local HIV prevention policy recommendations as well as support strategic planning and allocation to key population programmes.

In this paper, we estimate the potential impact of expanding PrEP coverage by offering CAB‐LA to MSM populations in high‐income countries based on models from three groups that accepted the invitation to participate in this model comparison project. We compared results from risk‐stratified HIV transmission dynamic models among MSM which were independently parameterized and calibrated to local data from Atlanta in the United States [17], Montreal in Canada [18] and in the Netherlands [19, 20], populations with HIV epidemics dominated by MSM transmission. Population‐level effectiveness and efficiency were estimated over 20 years and the feasibility of different PrEP expansion strategies for each epidemic setting is discussed.

## 2 METHODS

We present results from three models of the HIV epidemic in MSM populations (parameterized and calibrated for Atlanta, USA, Montreal, Canada, and the Netherlands) that simulated a set of pre‐designated PrEP expansion scenarios over 20 years (2022–2042) and provided inputs and results in the requested forms. Modelling teams were expected to employ estimates of TDF/FTC PrEP efficacy and adherence representative of the populations they simulated. The goal was to present a spectrum of potential outcomes associated with the expansion of PrEP coverage by increasing CAB‐LA usage and/or switching existing TDF/FTC PrEP users to CAB‐LA [17, 18, 19, 20]. CAB‐LA efficacy (91%, range 82%–96%) and retention rates (16.8% discontinuation over 12 months) from the HPTN 083 trial were standardized across the models [1], while TDF/FTC effectiveness (incorporating adherence), population access and propensity to use PrEP, and other parameters, were specific to each model and its geographic setting. All models reported results with at least 100 calibrated parameter sets which best represent the HIV epidemic in their setting. The modelling assignment provided to the participating teams is included in the Supporting Information.

### 2.1 Participating teams and settings

**Figure 1:**
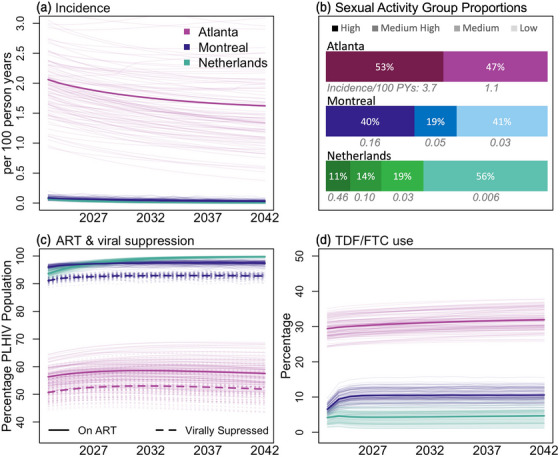
Baseline model characteristics in Atlanta, Montreal and the Netherlands. (A) HIV incidence. (B) Proportion of the population and estimated HIV incidence in each sexual activity group in 2022. (C) Antiretroviral therapy (ART) and viral suppression in the PLHIV population (viral suppression was not simulated in the Netherlands model). (D) Projected tenofovir disoproxil and emtricitabine (TDF/FTC) use among MSM not living with HIV. Bold lines show means of all scenario replicates. Thin lines show individual simulations. Variation shown in the range of simulation lines comes from parameter variability in the deterministic compartmental models and stochastic variability in the agent‐based model. Abbreviations: PLHIV, people living with HIV; MSM, men who have sey with men

The Atlanta model developed by the HPTN Modelling Centre [17] was used to assess the impact of interventions in an MSM population with high HIV prevalence. The Montreal [18] and Netherlands [19, 20] models, developed by the McGill University and Erasmus Medical Centre/Maastricht University teams, respectively, reflected settings with lower HIV prevalence among MSM. Table 1 summarizes key characteristics of each model and Figure 1 shows baseline population characteristics and risk configurations assumed in each model. More details and parameter tables for each model are included in the Supporting Information.

**Table 1:** Model characteristics

Model characteristics	Atlanta	Montreal	Netherlands
Model description	Deterministic compartmental	Stochastic agent‐based	Deterministic compartmental
Population stratification	Age (18–24, 25+ years old), race (Black, White), sexual activity group (PrEP indication)	Age (15–24, 25–34, 35–44, 45–54, ≥ 55), sexual activity group (≤5, 6–10, ≥11 anal sex partners per year)	Sexual activity group (IQR^a^ <1, 2–4, 8–12, 35–55 new partners every 2 years)
Age range	18+	15+	15+
TDF/FTC effectiveness	82% (range: 75%–87%) [9, 21]	86% [22]	85% [10]
TDF/FTC discontinuation rate	0.33/year [21]	0.90/year [23] (0.84–0.96 IQR)^b^	0.62/year (0.48–0.79 IQR)^b^
CAB‐LA effectiveness*	91% (82%–96% range) [1]		
CAB‐LA discontinuation rate*	0.168/year [1]		
Proportion in each sexual activity group^c^	53% high 47% low	40% high 19% medium 41% low	11% (11%–12% IQR) high 14% (12%–16% IQR) medium high 19% (14%–23% IQR) medium 56% (49%–61% IQR) low^b^
HIV incidence ratio between sexual activity groups^d^	High sexual activity group 3.3x risk of low sexual activity group	High sexual activity group 5–7x that of lower sexual activity group (after 1985)	High sexual activity group 80x risk of low sexual activity group (58–116 IQR) Medium high sexual activity group 17x risk of low sexual activity group (12–28 IQR) Medium sexual activity group 5x risk of low sexual activity group (3–7 IQR)^b^
Median HIV incidence in each sexual activity group^d^	High: 3.7/100 person‐years (PYs) Low: 1.1/100 PYs	High: 0.16/100 PYs Medium: 0.05/100 PYs Low: 0.03/100 PYs	High: 0.46/100 PYs Medium‐high: 0.10/100 PYs Medium: 0.03/100 PYs Low: 0.006/100 PYs
PrEP allocation by sexual activity group (PrEP‐eligible model scenario)	Cover 90% of high sexual activity group, then offer to low sexual activity group	Each individual was assessed for eligibility and a binomial distribution with a probability of uptake determined if those eligible initiated PrEP^e^	Cover the two highest sexual activity groups, then offer to lower sexual activity groups

^a^ Interquartile range.

^b^ Following model calibration.

^c^ On 1 January 2022.

^d^ Annual HIV incidence between groups in the absence of PrEP.

^e^ Coverage by risk group 1 January 2022: 4% (IQR: 3%–5%) low activity group; 7% (IQR: 5%–9%) medium activity group; 9% (IQR: 7%–12%) high activity group.

* Constant between all models.

Abbreviations: TDF/FTC, tenofovir disoproxil fumarate/emtricitabine; CAB‐LA, long‐acting injectable cabotegravir; PrEP, pre‐exposure prophylaxis.

### 2.2 Baseline scenarios with TDF/FTC use only

Baseline scenarios were simulated to project TDF/FTC use and the HIV care continuum in each setting in the absence of CAB‐LA and serve as counterfactual estimates when evaluating CAB‐LA expansion scenarios. Parameters related to antiretroviral therapy (ART) use, including initiation and discontinuation rates, and rates of achieving viral suppression (VS) were based on local data in the simulated communities between 2022 and 2042. Model projections of TDF/FTC use, initiated in accordance with local PrEP eligibility criteria, used availability and acceptability measures set to reflect current and expected future use.

A comparison of the predicted epidemic under the baseline scenario across models is presented in Figure 1 and Table S1. The predicted HIV prevalence and incidence in 2022 were significantly higher in the Atlanta model (26% and 2.1 per 100 PYs) than in the Montreal model (6% and 0.1 per 100 PYs) or the Netherlands model (9% and 0.1 per 100 PYs). HIV incidence was predicted to decrease by 21% (Atlanta), 63% (Montreal) and 98% (Netherlands) from 2022 to 2042 even without offering CAB‐LA. The estimated HIV treatment cascade in 2022 was better in Montreal and the Netherlands than in Atlanta with 95% (Montreal), 94% (Netherlands) and 56% (Atlanta) of MSM living with HIV on ART and 90% (Montreal) and 51% (Atlanta) of MSM living with HIV virally suppressed. VS in the Netherlands is very high (96% [24]) and was not included explicitly in that model. The proportions of MSM living with HIV on ART and virally suppressed were expected to improve slightly over time in all settings. The Netherlands model assumed the fewest PrEP‐eligible MSM in 2022 (26% of MSM not living with HIV [12]) compared to Atlanta (47% of MSM not living with HIV) or Montreal (59% of MSM not living with HIV). The Atlanta model had substantially higher TDF/FTC use among MSM not living with HIV (29%) in 2022 compared to 6% in Montreal and 4% in the Netherlands. Sexual activity groups varied between models; 53% (Atlanta), 40% (Montreal) and 25% (the Netherlands) of MSM were in the high or medium‐high sexual activity groups.

### 2.3 PrEP expansion scenarios

**Figure 2:**
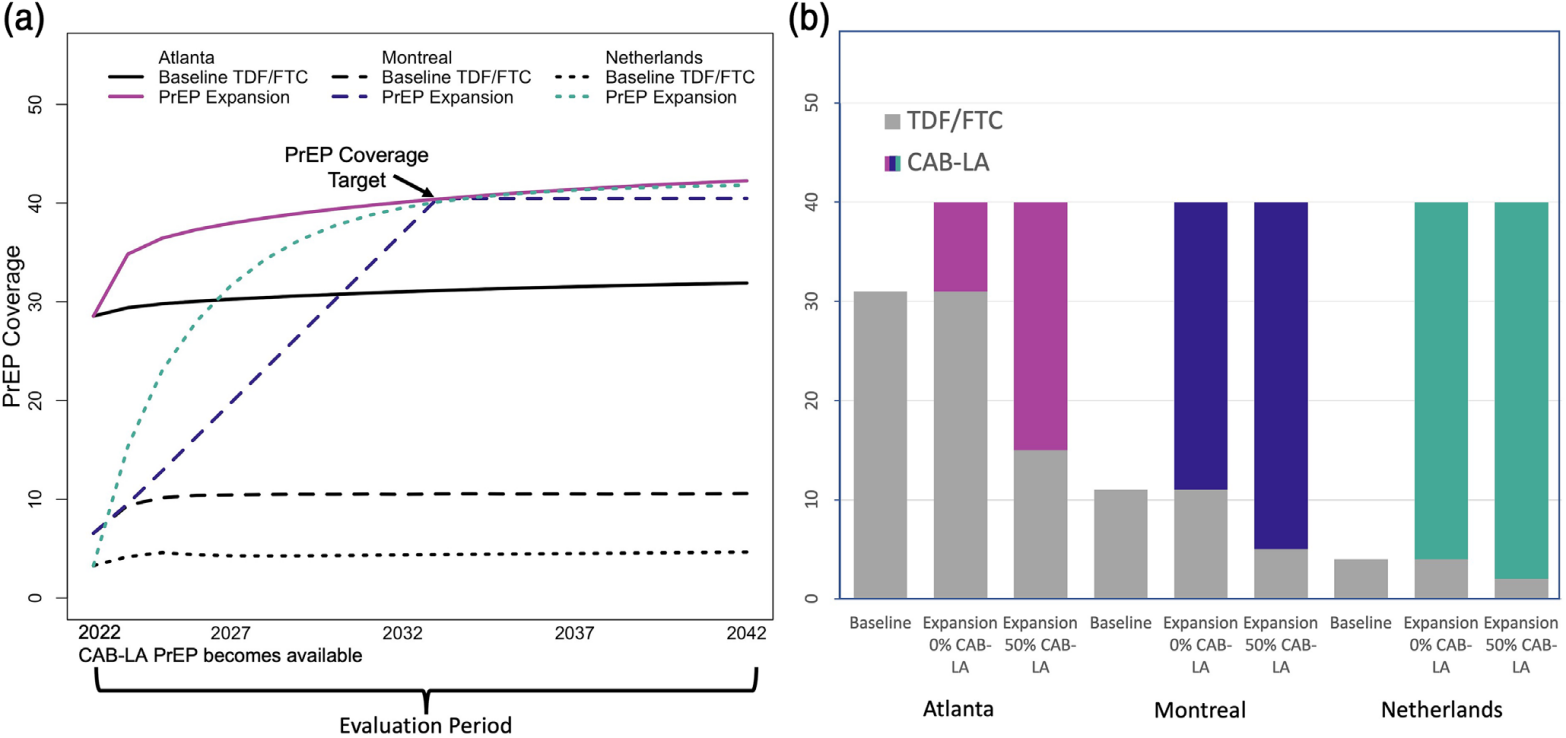
Pre‐exposure prophylaxis (PrEP) expansion scenario structure. (A) The baseline (counterfactual) scenario projected that tenofovir disoproxil and emtricitabine (TDF/FTC) usage will increase slightly over time from its current level in 2022 (29% Atlanta, 6% Montreal and 4% Netherlands) to 2042 (32% Atlanta, 10% Montreal and 5% Netherlands, black lines). Reaching a targeted coverage (40%) in 10 years (by 2032) required initiating new long‐acting cabotegravir (CAB‐LA) users (coloured lines). (B) The distribution of CAB‐LA and TDF/FTC use in each scenario depended on the prevalence of TDF/FTC use in the baseline scenario. Expansion with 0% CAB‐LA use caused only those in the expansion group to use CAB‐LA. Expansion with 50% CAB‐LA caused 50% of the baseline TDF/FTC users to instead use CAB‐LA, in addition to those in the expansion group who always use CAB‐LA.

CAB‐LA expansion was initiated in 2022 and increased the overall PrEP coverage (including both TDF/FTC and CAB‐LA users) to between 15% and 50% of the total population of MSM not living with HIV (Table 2 and Figure 2). Two potential PrEP coverage timelines were included, one in which coverage targets were expected to be reached by 1 January 2027 (5‐year target) and one by 1 January 2032 (10‐year target). PrEP coverage could not exceed the targeted coverage by more than 1 percentage point (pp). The analysis was restricted to scenarios where the targeted coverage following CAB‐LA expansion exceeded the projected PrEP coverage in the baseline scenario. Different proportions (0%–100%, Table 2) of TDF/FTC users were assumed to switch to CAB‐LA at the start of the simulation (1 January 2022) and the same proportion of baseline TDF/FTC users were assumed to initiate CAB‐LA instead of TDF/FTC throughout the simulation (Figure 1). Recruitment of new CAB‐LA users (in addition to those projected to switch from TDF/FTC) was modelled in two different ways: (i) preferentially from groups of PrEP‐eligible MSM at higher risk as defined in each model and (ii) proportionally across the population assuming universal access to CAB‐LA.

**Table 2:** PrEP expansion intervention details

Intervention components	Scenarios simulated
PrEP coverage of MSM not living with HIV (TDF/FTC + CAB‐LA PrEP users combined)	15%, 30%, 40% and 50%
Time to achieve targeted PrEP coverage	5 and 10 years (by 2027 and by 2032)
Proportion of current/projected TDF/FTC users switching to CAB‐LA	0%, 15%, 30%, 50% and 100%
Recruitment of new CAB‐LA users (users in addition to those on TDF/FTC PrEP at baseline)	Based on current PrEP eligibility criteria: those in higher sexual activity groups prioritized for PrEP coverage Proportionally distributed: distributed across risk and age groups to all MSM not living with HIV

Abbreviations: PrEP, pre‐exposure prophylaxis; MSM, men who have sex with men; TDF/FTC, tenofovir disoproxil and emtricitabine; CAB‐LA, long‐acting cabotegravir.

### 2.4 Metrics of impact

Population‐level effectiveness was assessed by two metrics: (i) the *cumulative fraction of new HIV acquisitions averted over 20 years* (calculated as the complement of the cumulative number of new HIV acquisitions between 2022 and 2042 in each PrEP expansion scenario divided by the cumulative number of HIV acquisitions in the baseline scenario) and (ii) the *relative reduction in annual HIV incidence after 10 years* (calculated as one minus the incidence in 2032 divided by the baseline HIV incidence in 2022). To better understand the projected impact of PrEP expansions, we calculated the “*effective risk coverage*” based on the proportion of MSM in each sexual activity group, HIV incidence ratio between groups and PrEP coverage achieved in each group as reported in Table 1. This measured the proportion of HIV risk covered when targeted coverage is reached in 2032 calculated from PrEP coverage of each sexual activity group weighted by the proportion of new acquisitions expected to occur in that group. Sexual activity group size and incidence were calculated from group proportions and incidence at baseline on 1 January 2022. Population‐level efficiency was measured as the *number needed to treat (NNT)* to prevent one HIV acquisition calculated as the number of additional PYs on PrEP in each PrEP expansion scenario (compared to baseline) divided by the number of acquisitions averted (difference from baseline) between 2022 and 2042. Unless otherwise stated, all metrics are the medians over the full range of switching scenarios for each PrEP expansion level.

## 3 RESULTS

### 3.1 Expanding PrEP coverage with CAB‐LA among MSM at higher risk

**Figure 3:**
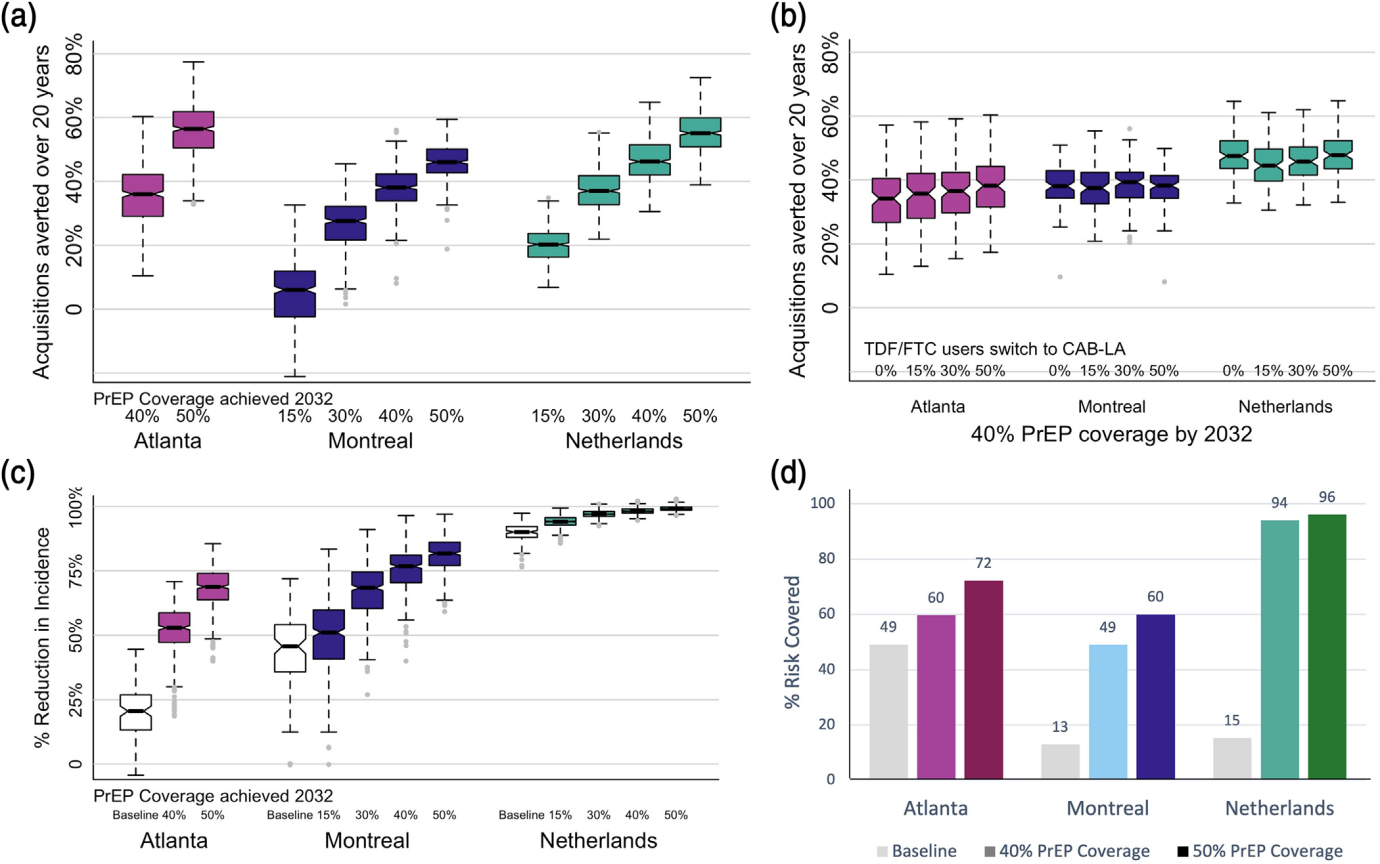
Population effectiveness. (A) Acquisitions averted over 20 years (2022–2024) with pre‐exposure prophylaxis (PrEP) coverage achieved by 2032. Fifteen percent and 30% PrEP coverage levels were not modelled in Atlanta as baseline PrEP coverage was 29%. (B) Acquisitions averted with 40% PrEP coverage achieved by 2032 for different proportions of tenofovir disoproxil and emtricitabine (TDF/FTC) users switching to long‐acting cabotegravir (CAB‐LA). (C) Projected reductions in HIV incidence in 2032 relative to 2022 incidence in the baseline (white) and expanded PrEP coverage (colours) scenarios. A and C cumulate all TDF/FTC to long‐acting cabotegravir (CAB‐LA) switching scenarios. (D) Proportion of risk covered for different PrEP expansions, estimated by the proportion of PrEP coverage of each sexual activity group weighted by the proportion of new acquisitions expected to occur in that group. Notches in boxplot show 95% credible interval for the median. Dotted lines show full results range without outliers.

Increasing overall PrEP coverage by 11 pp to 40% by 2032 in Atlanta was expected to avert median 36% of new acquisitions over 20 years when higher sexual activity groups were prioritized (Figure 3A). Substantially larger increases in PrEP coverage (33 and 26 pp to achieve 40% and 30% coverage in Montreal and the Netherlands, respectively) were needed to avert a similar fraction of new acquisitions (38% in Montreal and 37% in the Netherlands). Increasing overall PrEP coverage by an additional 10 pp by 2032 to 50% in Atlanta and Montreal and 40% in the Netherlands resulted in an additional 20 pp acquisitions averted in Atlanta, 8 pp in Montreal and 9 pp in the Netherlands. Meeting coverage targets 5 years earlier (by 2027) resulted in averting 2–5 pp more acquisitions in Atlanta across expansion scenarios compared to 3–10 pp more in Montreal and 5–6 pp more in the Netherlands (full 5‐year coverage results shown in Figures S1–S3).

Increasing the proportion of TDF/FTC users switching to CAB‐LA (Figure 3B and Figures S4–S7) had only a small positive effect in Atlanta (4 pp) and almost no effect in Montreal and the Netherlands, provided that the same overall PrEP coverage is reached. The proportions of TDF/FTC users who could potentially switch to CAB‐LA depended on the proportion of current and projected PrEP users in the baseline, which varied across models as baseline PrEP use ranged from 29% in Atlanta to 7% in Montreal and 4% in the Netherlands.

Population effectiveness, measured as a relative reduction in annual HIV incidence, strongly depended on the expected incidence reduction in baseline scenarios. In Atlanta and Montreal, where the projected baseline reduction in incidence was low or moderate, expansions to 40% PrEP coverage were expected to reduce HIV incidence by an additional 32 and 31 pp, respectively (Figure 3C). In the Netherlands, where the projected baseline reduction in incidence was high, reaching 40% PrEP coverage was only expected to reduce HIV incidence by an additional 8 pp.

The expected coverage of HIV risk at each PrEP coverage level varied greatly between models (Figure 3D). In Atlanta, baseline risk coverage was high at 49% while reaching 40% PrEP coverage by 2032 covered 60% of the risk. At baseline, Montreal and the Netherlands both had comparably low‐risk coverage, (13% and 15%, respectively), but expansion to 40% PrEP coverage by 2032 resulted in significantly lower risk covered in Montreal (49%) compared to the Netherlands (94%).

**Figure 4:**
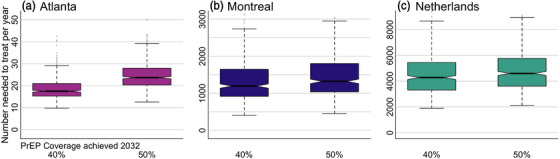
Population efficiency. Additional years on pre‐exposure prophylaxis (PrEP) needed to prevent one HIV acquisition with PrEP coverage achieved by 2032 in (A) Atlanta, (B) Montreal and (C) the Netherlands. Includes all tenofovir disoproxil and emtricitabine (TDF/FTC) to long‐acting cabotegravir (CAB‐LA) switching scenarios. Note different y‐axes. Notches in boxplot show 95% credible interval for the median. Dotted lines show full results range without outliers.

Projected population efficiency strongly depended on the HIV incidence over the 2022–2042 period (Figure 4). The PrEP expansion in Atlanta, where HIV incidence in the baseline scenario remained high (between 1.5% and 2%), was highly efficient with an estimated NNT of 17 and 24 with 40% and 50% PrEP coverage achieved by 2032, respectively. In comparison, the projected NNT in Montreal, where annual HIV incidence was below 0.1% in the baseline scenario, was substantially higher at 1194 (40% PrEP coverage by 2032) and 1320 (50% PrEP coverage by 2032). The corresponding NNT was estimated to be greater than 4000 for the Netherlands where the existing HIV prevention programmes were expected to practically eliminate HIV transmission by 2042 in the baseline.

### 3.2 Impact of PrEP expansion on racial disparities

**Figure 5:**
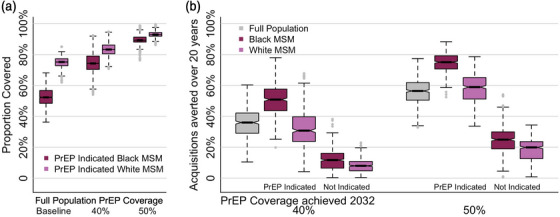
Effects of pre‐exposure prophylaxis (PrEP) expansion on racial disparities in the Atlanta model. (A) Racial differences in the proportion of men who have sex with men (MSM) with PrEP indications on PrEP with baseline and expanded PrEP coverage. Median PrEP coverage of MSM with no PrEP indication was 0% in all scenarios. (B) Acquisitions averted over 20 years in Black and White MSM with and without a PrEP indication. Notches in boxplot show 95% credible interval for the median. Dotted lines show full results range without outliers.

PrEP expansion with CAB‐LA represents an opportunity to begin to rectify the racial disparities that have thus far existed in the distribution of TDF/FTC PrEP [11]. The Atlanta model was the only model in this comparison to include race; here, 62% of modelled individuals were Black MSM and 38% were White MSM. Fifty‐three percent of modelled Black MSM and 55% of modelled White MSM had a PrEP indication. Baseline PrEP coverage reflected current disparities in PrEP access as PrEP‐indicated White MSM had 23 pp higher PrEP coverage than PrEP‐indicated Black MSM. In comparison, PrEP expansion with 40% and 50% coverage by 2032 significantly reduced this difference to 9 and 4 pp, respectively (Figure 5A). Distributing additional PrEP coverage in these expansions to previously uncovered MSM with PrEP indications is expected to narrow the PrEP usage gap by race due to substantially higher proportions of acquisitions averted in PrEP‐indicated Black MSM (50% and 75%) with 40% and 50% overall PrEP coverage by 2032, respectively (Figure 5B). The NNT was significantly lower in PrEP‐indicated Black MSM than PrEP‐indicated White MSM, with NNT of 17 and 70, respectively, with 40% overall PrEP coverage by 2032 (Figure S8).

### 3.3 Expanding PrEP coverage with CAB‐LA available to all MSM

**Figure 6:**
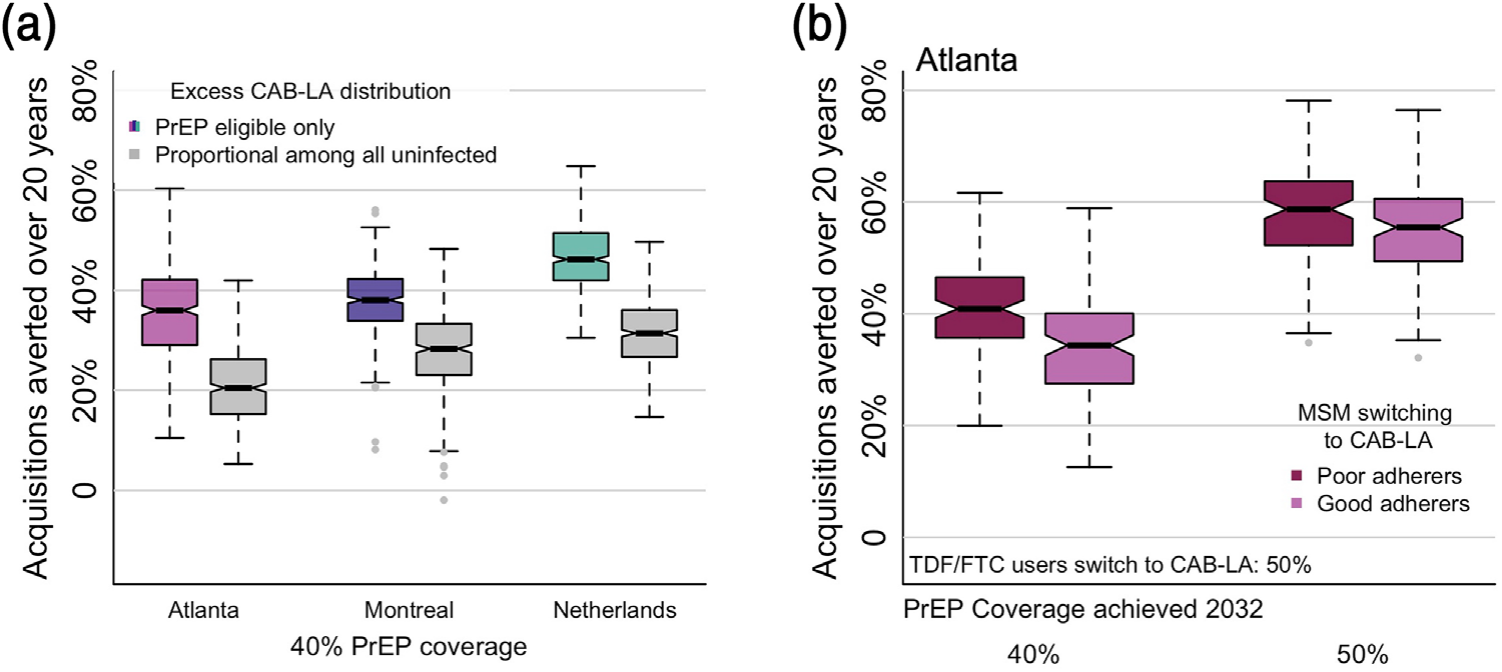
Additional pre‐exposure prophylaxis (PrEP) expansion scenarios. (A) PrEP expansion among PrEP‐eligible men who have sex with men (MSM) versus proportionally among all MSM not living with HIV. Acquisitions averted when PrEP expansion is only to those PrEP‐eligible (colours) or proportionally among all MSM not living with HIV (grey) with 40% PrEP coverage, including all tenofovir disoproxil and emtricitabine (TDF/FTC) to long‐acting cabotegravir (CAB‐LA) switching scenarios. (B) Prioritized interventions based on adherence to TDF/FTC in the Atlanta model. Poor TDF/FTC adherers switching to CAB‐LA (darker colours) and good TDF/FTC adherers switching to CAB‐LA (lighter colours) with 40% and 50% PrEP coverage achieved by 2032. Notches in boxplot show 95% credible interval for the median. Dotted lines show full results range without outliers.

Providing universal access to CAB‐LA was modelled by expanding PrEP coverage proportionally throughout the entire population, instead of offering it preferentially to MSM at higher risk, as a hypothetical comparison to reflect when PrEP programmes are not well prioritized to those at risk. Such an intervention was projected to have a lower impact in terms of percent of acquisitions averted with 40% PrEP coverage by 2032 by a median 16 pp in Atlanta, 10 pp in Montreal and 15 pp in the Netherlands (Figures 6A and S9). This also made PrEP expansions in the Atlanta model less equitable. Significantly larger disparities in PrEP coverage by race (16 and 14 pp) remained as coverage increased to 40% and 50%, respectively, in the proportional expansion compared to the expansion prioritizing those in higher sexual activity groups, with 28 and 43 pp fewer acquisitions averted among PrEP‐indicated Black MSM (Figure S10).

### 3.4 Importance of adherence in who is choosing CAB‐LA over TDF/FTC

In all simulations thus far, the models assumed that the group who switched to CAB‐LA was representative of all TDF/FTC users. Further analyses were performed with the Atlanta model, in which CAB‐LA expansion was either (i) successfully prioritized to poor TDF/FTC adherers, which resulted in improved PrEP effectiveness (90%) in those who remained on TDF/FTC or (ii) alternatively, prioritized to good TDF/FTC adherers, which resulted in reduced PrEP effectiveness (60%) among those who remained on TDF/FTC (Figure 6B). When 50% of TDF/FTC users switched to CAB‐LA, adherence prioritizing improved impact by 7 pp with 40% PrEP coverage. The importance of adherence prioritizing decreased with higher PrEP coverage levels.

## 4 DISCUSSION

Our analysis focused on the expected benefits of adding CAB‐LA to the HIV prevention portfolio in populations with HIV epidemics dominated by MSM transmission assuming that PrEP expansion with CAB‐LA will improve both overall PrEP coverage and PrEP adherence. Our findings suggest that expanding CAB‐LA may avert a significant proportion (36%) of expected new HIV acquisitions over the next 20 years in a setting where TDF/FTC coverage and HIV incidence are both high (like Atlanta) if the expansion increases the current PrEP coverage in the MSM population by a third. However, a substantially larger increase in overall PrEP coverage (double or triple the current coverage) would be needed to achieve a comparable reduction of acquisitions averted in settings with low current PrEP use and HIV incidence (like Montreal and the Netherlands). The Netherlands model was able to cover nearly all HIV risk with 50% PrEP coverage leading to an almost 100% reduction in incidence. Notably, all models predicted that the number of expected HIV acquisitions over 20 years will be reduced by close to 60% if 50% population PrEP coverage is reached by 2027. However, this result might not be generalizable to other settings given this metric's dependence on multiple factors, such as differences in PrEP eligibility, initial PrEP coverage and expected PrEP use in the baseline scenarios in which only TDF/FTC is available. On the other hand, all PrEP expansion scenarios included in this analysis may still present quite reasonable approximations of the expected intervention impact, even if the increased PrEP coverage is achieved with the addition of other, even slightly better PrEP products (as PrEP efficacy cannot improve greatly above the 91% assumed here) over the 20‐year period.

We found that expanding the PrEP toolbox with CAB‐LA is projected to be a highly efficient intervention in places with high HIV incidence (like Atlanta) where the estimated additional years on PrEP needed to prevent one acquisition (NNT) were expected to be comparable to TDF/FTC interventions among adult MSM [25] and among Black adolescent sexual minority males [26], and significantly more efficient than the daily TDF/FTC interventions tested in the iPrEx study [27]. In contrast, the estimated efficiency of PrEP expansions was low in Montreal and the Netherlands, mainly due to the low and decreasing HIV incidence in these populations—even in the baseline scenario—leaving few acquisitions to be prevented by any new intervention.

The Atlanta model showed that CAB‐LA PrEP expansions that manage to reach the majority of MSM with PrEP indications could lead to more equitable outcomes. Baseline PrEP coverage reflected current PrEP disparities [11], while increased overall PrEP coverage led to both much higher PrEP coverage and to high proportions of acquisitions averted among Black MSM. Addressing this unmet need for PrEP by expanding CAB‐LA coverage in Black MSM could help address the continuing racial disparities in HIV incidence. However, the success of such interventions depends on the ability to reach and engage MSM who face barriers to PrEP access or currently do not consider PrEP as a viable prevention option.

Recruiting new CAB‐LA PrEP users had a larger impact than switching existing PrEP users from TDF/FTC to CAB‐LA. With 40% PrEP coverage, adding PrEP users from the high sexual activity groups increased acquisitions averted substantially in each model but the proportion of baseline PrEP users switching to CAB‐LA did not, due to the high effectiveness (combining efficacy and adherence) to TDF/FTC assumed in the models. This finding agrees with the results from another model of CAB‐LA use by MSM in the southeastern United States [28]. This study found that increasing PrEP coverage with CAB‐LA would avert more acquisitions than switching existing users with 17% of acquisitions averted over 10 years if CAB‐LA caused the PrEP initiation rate to double, compared to only 4% if half of the existing TDF/FTC users switched to CAB‐LA.

Other published models of CAB‐LA in MSM populations have focused on scenarios in which only TDF/FTC or CAB‐LA was available. This precludes these models from estimating the effects of switching between regimens or the population impacts when multiple regimens are in use. One analysis of offering PrEP to MSM and transgender women at very high risk of HIV acquisition in the United States showed relatively small differences in acquisitions averted if all individuals used CAB‐LA (68%) versus TDF/FTC (60%) [29]. A larger difference in the reduction in new HIV acquisitions (11 pp) achieved with 35% PrEP coverage of CAB‐LA, compared to TDF/FTC was projected with an agent‐based model of MSM in Atlanta as a result of larger difference in the products’ assumed effectiveness [30].

This analysis has several limitations. The effectiveness (which incorporates both efficacy and adherence) of TDF/FTC, informed by local data in all three models, was assumed to be high, which may underestimate the advantage of CAB‐LA over TDF/FTC. Therefore, these results are not generalizable to populations with lower TDF/FTC adherence. Conservatively, the Atlanta and Netherlands models assumed no protection in the cabotegravir tail phase after CAB‐LA discontinuation. This assumption should be reassessed after the completion of ongoing open‐label extensions of the CAB‐LA trials [1, 2]. While one model incorporated race, modelled CAB‐LA expansions used current local guidance to recommend PrEP based on sexual behaviour only. Models assumed different discontinuation rates for TDF/FTC and CAB‐LA, which may not reflect true behaviour, as discontinuation rates were similar for both TDF/FTC and CAB‐LA in the HPTN 083 trial (although all trial participants received both oral tablets and injections) [1]. ART use and VS levels were not varied between simulations, precluding insights into the effect of changing ART coverage with rising PrEP coverage.

Adding long‐acting injectable PrEP as an HIV prevention option for MSM has several important implications. First, the effectiveness of CAB‐LA was demonstrated against a highly effective competitor (TDF/TFC) in clinical trials [1, 2], suggesting that it is reasonable to associate it with improved adherence. Second, offering a completely different and less frequent delivery route may appeal to new users and result in higher overall PrEP coverage. In preference surveys, 73% of MSM reported they would be interested in long‐acting injectable PrEP [31]; young adults [32], young adult Black MSM [33] and MSM with higher self‐perception of risk [34] have also expressed preference for long‐acting injectable PrEP options. It is unclear if and how this preference will shape the future rollout of CAB‐LA and other long‐acting PrEP products but data from contraception studies have shown that more options lead to more users [35]. Thirty‐one percent of MSM currently taking oral TDF/FTC expressed preference for a long‐acting injectable in one survey, pointing to the likelihood of some users switching PrEP regimens when new options become available [14]. Third, offering new PrEP options to MSM with PrEP indications may help reduce HIV disparities if it leads to a significant increase in PrEP coverage in this group. When given a choice, the vast majority of MSM (over 95%) in the ongoing HPTN 083 open‐label extension decided to continue on CAB‐LA [36]. Finally, CAB‐LA serves as a proof of concept that long‐acting prevention solutions against HIV are possible, which should encourage the development of more products with different delivery routes and decreased frequency of use.

## 5 CONCLUSIONS

Our analysis suggested that expanding PrEP coverage with CAB‐LA can help progress towards ending the HIV epidemic, provided that it increases PrEP coverage and supports better adherence. However, PrEP expansion needs to occur equitably across all groups impacted by HIV. In the United States, Black and Hispanic MSM have lower rates of PrEP initiation than White MSM, despite their higher HIV burden [11] and PrEP persistence was lower in PrEP recipients who were younger or Black [37]. Fewer days with pills taken and high discontinuation of oral PrEP in younger and more economically disadvantaged MSM [38] makes them good candidates for CAB‐LA expansion. Our analysis shows that engaging and offering CAB‐LA to current and prospective oral PrEP users who struggle to adhere to daily oral PrEP could noticeably increase the impact of the intervention. This could help reduce, not widen, equity gaps when CAB‐LA becomes widely available.

## COMPETING INTERESTS

The authors have no competing interests to declare.

## AUTHORS’ CONTRIBUTIONS

Conception of the research question: SES, DTD and M‐CB. Model development, parameterization, coding and simulation: KMM, M‐CB, MM and DTD (Atlanta model); CMD, RMM, YX and MM‐G (Montreal model); DvdV and HW (Netherlands model). Model comparison analysis, statistical analysis, figure & table creation: SES and BH. Drafting of the manuscript: SES, JH and DTD. Critical input into draft manuscript: all authors. Read and approved the final manuscript: all authors.

## FUNDING

Funding for SES, JH, KMM, MM, DJD, BH, RB, M‐CB and DTD was provided by the NIAID, grant number UM1 068617. Funding for CMD, RMM, YX and MM‐G was provided by the Canadian Foundation for AIDS Research and the Canadian Institutes of Health Research. MM‐G's research programme is funded by a Canada Research Chair (Tier 2) in Population Health Modeling. CMD is supported by a doctoral award from the Fonds de recherche du Québec—Santé (FRQS).

## Supporting information


Supporting Information

## Data Availability

The model results that support the findings of this study are available from the corresponding author upon reasonable request.
