# Supplementary material for: Population‐level impact of expanding PrEP coverage by offering long‐acting injectable PrEP to MSM in three high‐resource settings: a model comparison analysis
Source: J Int AIDS Soc. 2023 Jul 13;26(Suppl 2):e26109. doi: 10.1002/jia2.26109 (PMC10339001; doi:10.1002/jia2.26109)
Supplement: Supplementary file 1 — Supporting Information [file JIA2-26-e26109-s001.docx]

**Population-level impact of expanding PrEP coverage by offering long-acting injectable PrEP to MSM in 3 high resource settings: A model comparison analysis**

**Supplementary Appendix**

Sarah E Stansfield^1*^, Jesse Heitner^2^, Kate M Mitchell^3,4^, Carla M Doyle^5^, Rachael M Milwid^5^, Mia Moore^1^, Deborah J Donnell^1,6^, Brett Hanscom^1^, Yiqing Xia^5^, Mathieu Maheu-Giroux^5^, David van de Vijver^7^, Haoyi Wang^7,8^, Ruanne Barnabas^2^, Marie-Claude Boily^3^, Dobromir T Dimitrov ^1,6^

**1. Supplementary Figures**

1. **Results including PrEP coverage levels achieved in 2027**

**
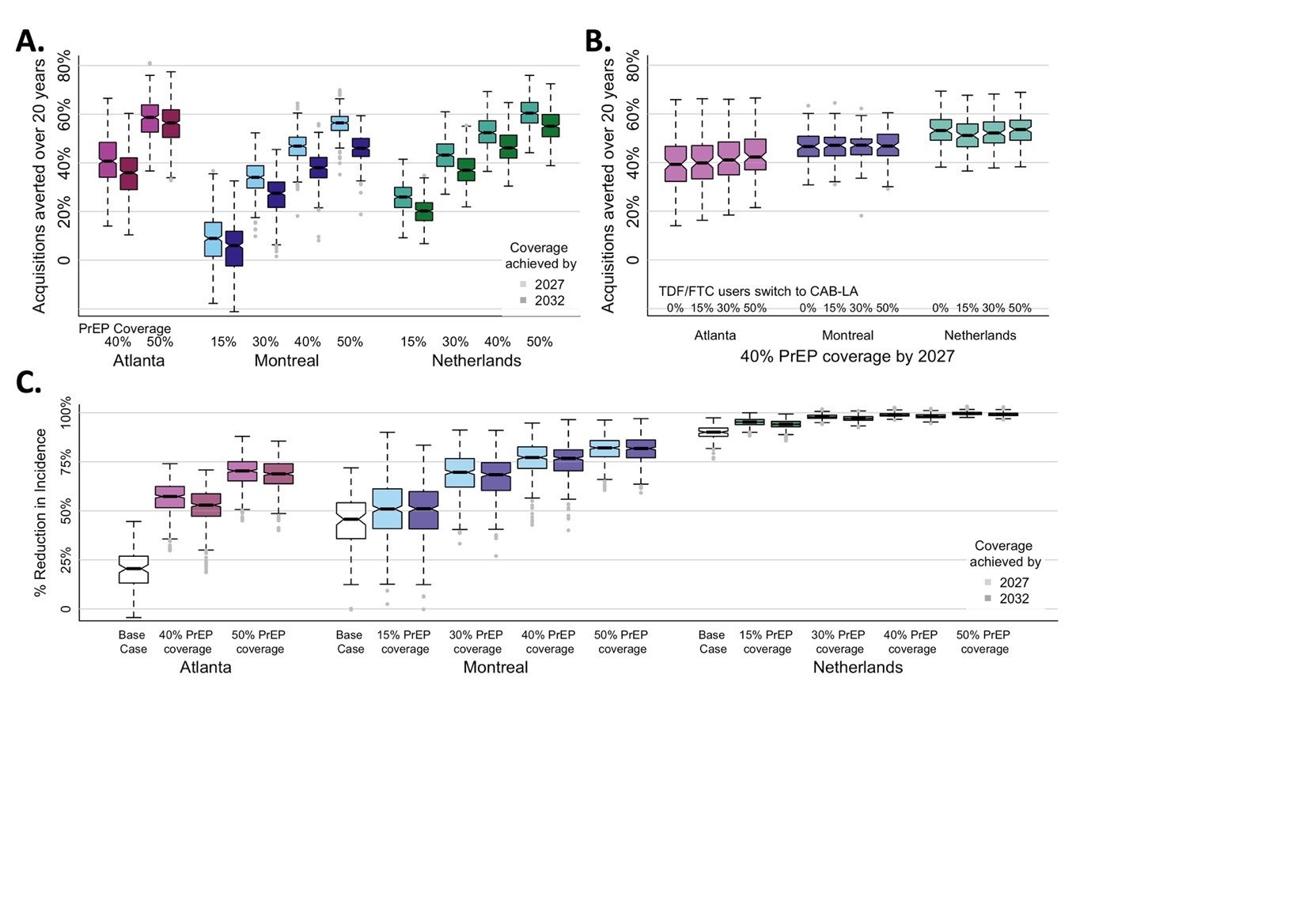
**

**Figure S1: Population effectiveness.** Projected acquisitions averted and incidence reduction from 2022-2042. A: Acquisitions averted over 20 years with PrEP coverage achieved by 2027 and 2032. 15 and 30% PrEP coverage levels were not modeled in Atlanta as baseline PrEP coverage was 29.4%. B: Acquisitions averted with 40% PrEP coverage by 2027 for different proportions of TDF/FTC users switching to CAB-LA. C: Projected reductions in HIV incidence in 2027 and 2032 relative to baseline 2022 incidence in the baseline (white) and expanded PrEP coverage (colors) scenarios. A & C include all TDF/FTC to CAB-LA switching scenarios. *Notches in boxplot show 95% credible interval for the median.*​ *Dotted lines show maximum/minimum without outliers.*​

**
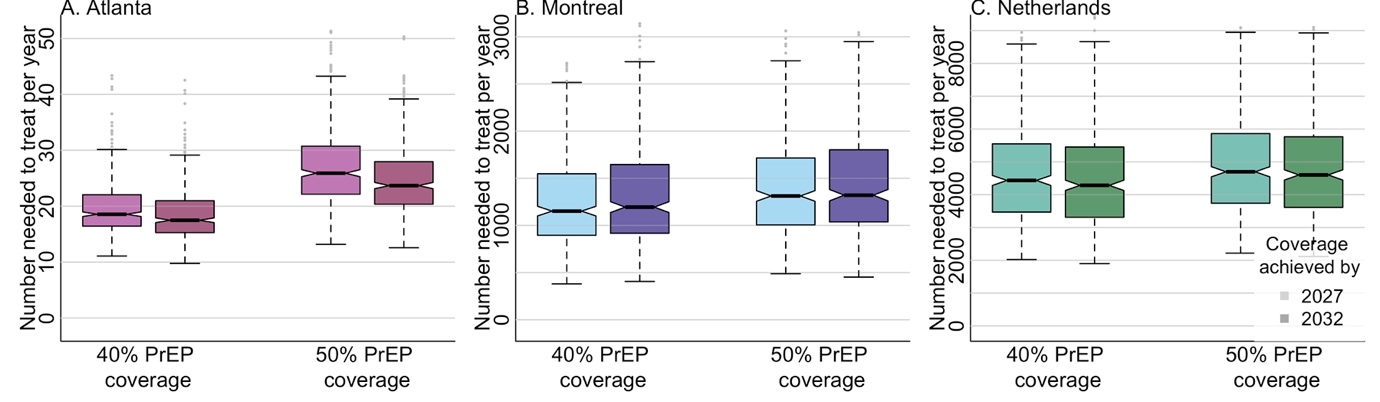
**

**Figure S2: Population efficiency.** Additional years on PrEP needed to prevent one HIV acquisition with PrEP coverage achieved by 2027 (lighter colors) and 2032 (darker colors) in A: Atlanta, B: Montreal, and C: the Netherlands. Includes all TDF/FTC to CAB-LA switching scenarios. *Note different y-axes.* *Notches in boxplot show 95% credible interval for the median.*​ *Dotted lines show maximum/minimum without outliers.*​

**
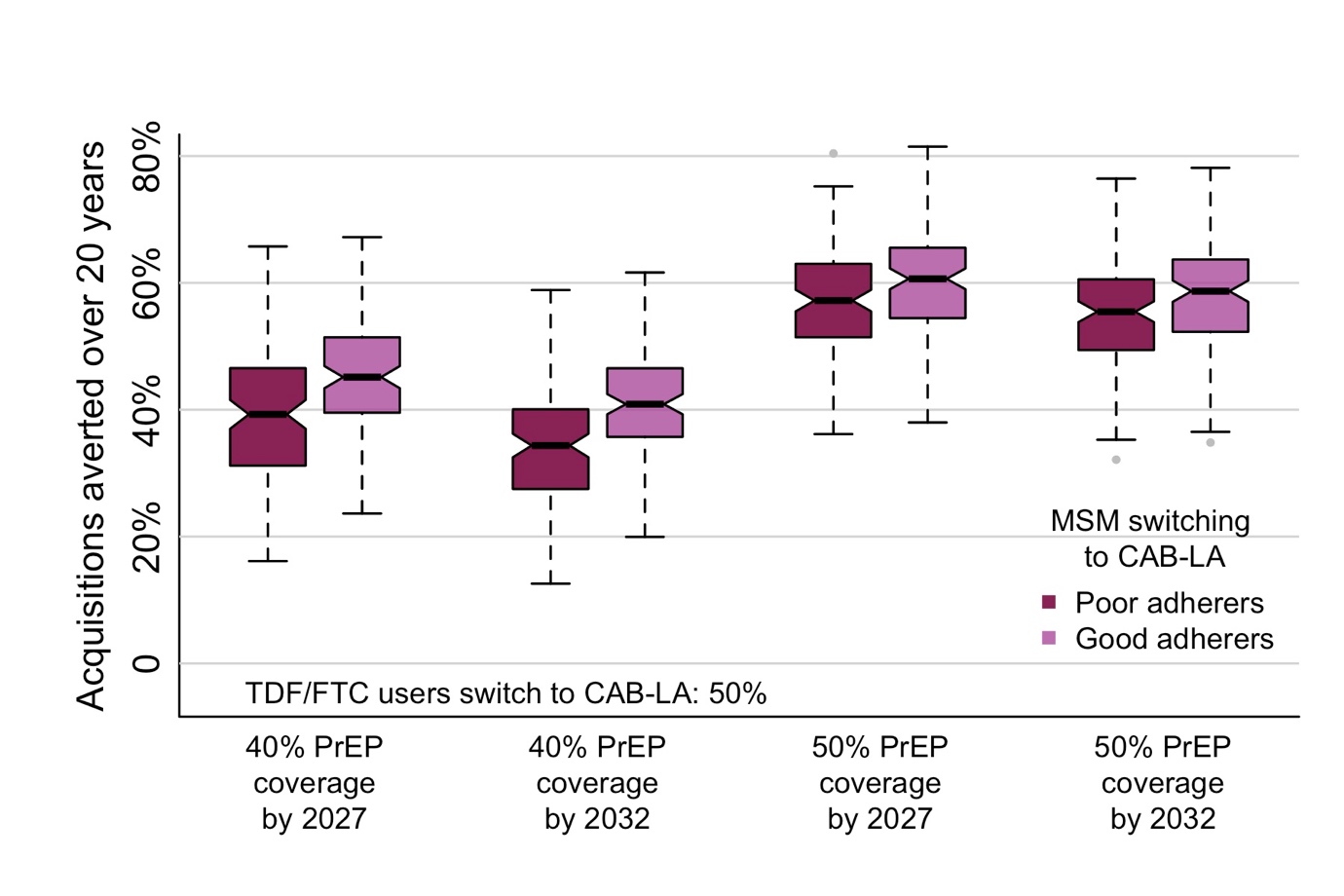
**

**Figure S3: Targeted interventions based on adherence to TDF/FTC.** Poor TDF/FTC adherers switching to CAB-LA (lighter colors) and good TDF/FTC adherers switching to CAB-LA (darker colors) with 40% and 50% PrEP coverage achieved by 2027 or 2032 in the Atlanta model. *Notches in boxplot show 95% credible interval for the median.*​ *Dotted lines show maximum/minimum without outliers.*​

**B. Results showing every level of TDF/FTC users switching to CAB-LA in each scenario**


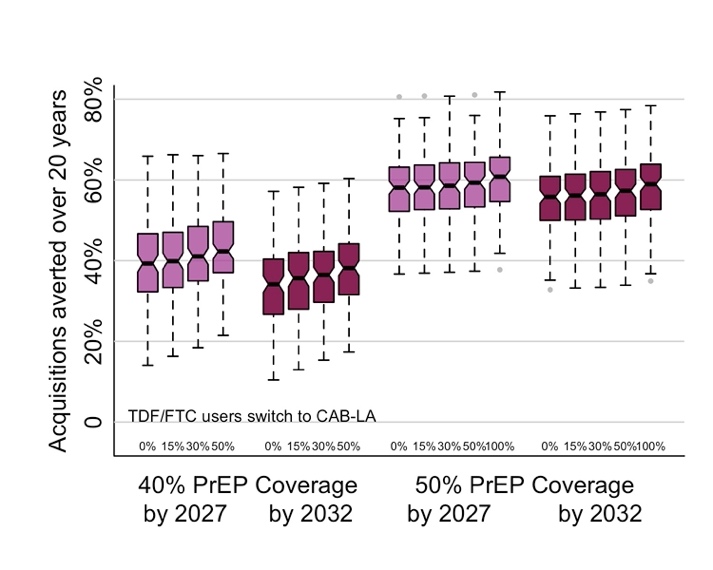


**Figure S4:** HIV acquisitions averted in Atlanta showing TDF/FTC users switching to CAB-LA at every PrEP coverage level.


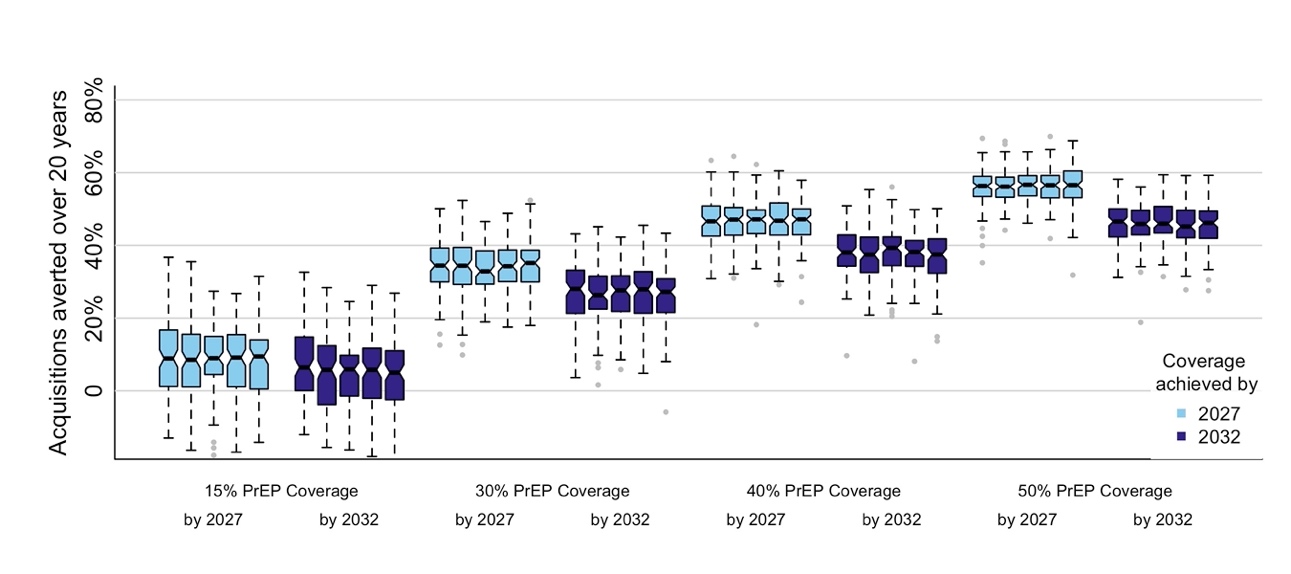


**Figure S5:** HIV acquisitions averted in Montreal showing TDF/FTC users switching to CAB-LA at every PrEP coverage level.


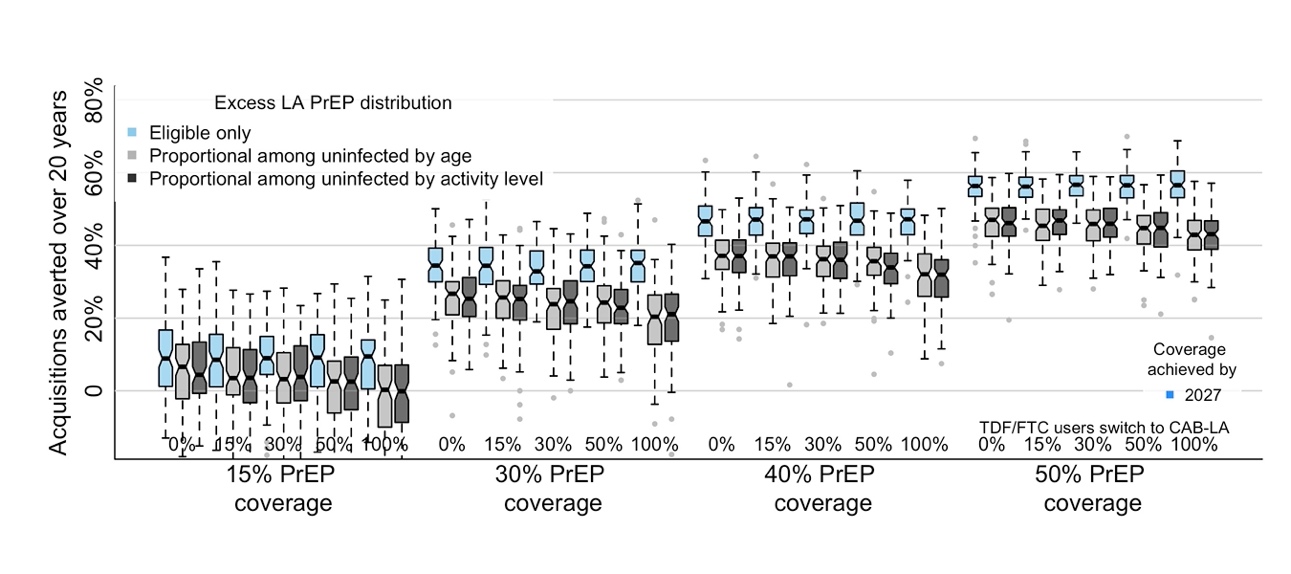


**Figure S6:** HIV acquisitions averted in Montreal with excess PrEP distribution in PrEP-eligible only (blue), proportionally among all uninfected by age (light gray), and proportionally among all uninfected by sexual activity level (dark gray).


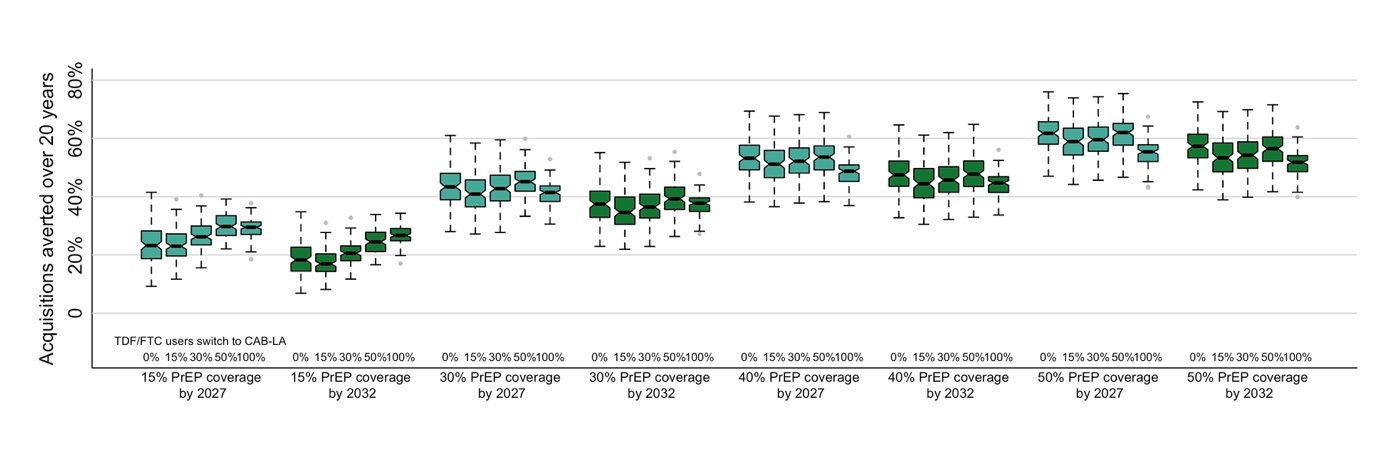


**Figure S7:** HIV acquisitions averted in the Netherlands showing TDF/FTC users switching to CAB-LA at every PrEP coverage level.

**C. Scenarios in showing the impact of PrEP expansion on racial disparities**

**
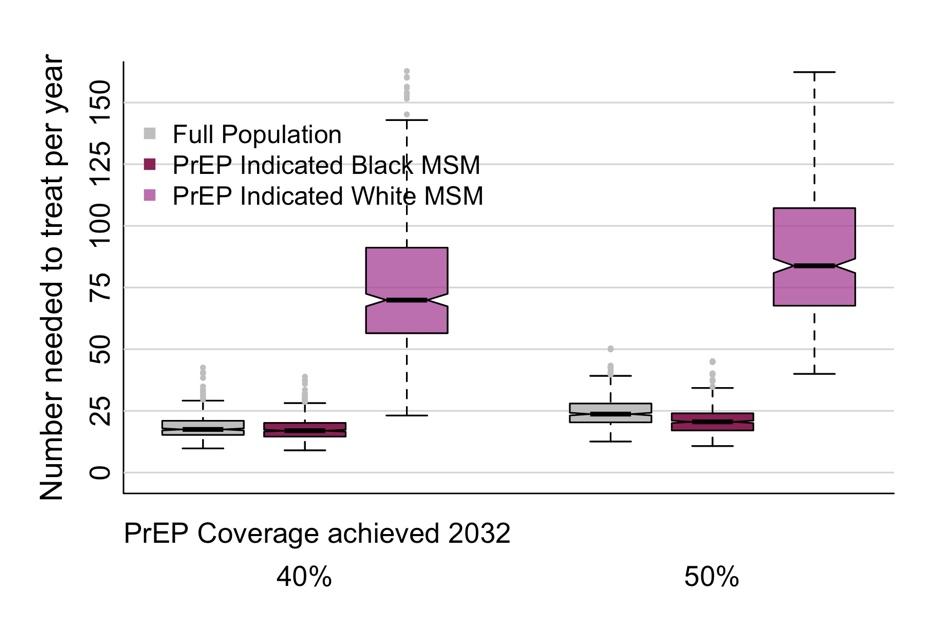
**

**Figure S8:** Racial differences in number needed to treat to prevent one HIV acquisition in PrEP-indicated Black MSM and White MSM in the Atlanta model. *Notches in boxplot show 95% credible interval for the median.*​ *Dotted lines show maximum/minimum without outliers.*

**D. Scenarios in which PrEP coverage was distributed proportionally through the population**

Switching from TDF/FTC to CAB-LA was more impactful in Atlanta and the Netherlands when excess CAB-LA was distributed proportionally among all those not living with HIV than when it was distributed to higher risk MSM preferentially, while there was little impact in Montreal.

**
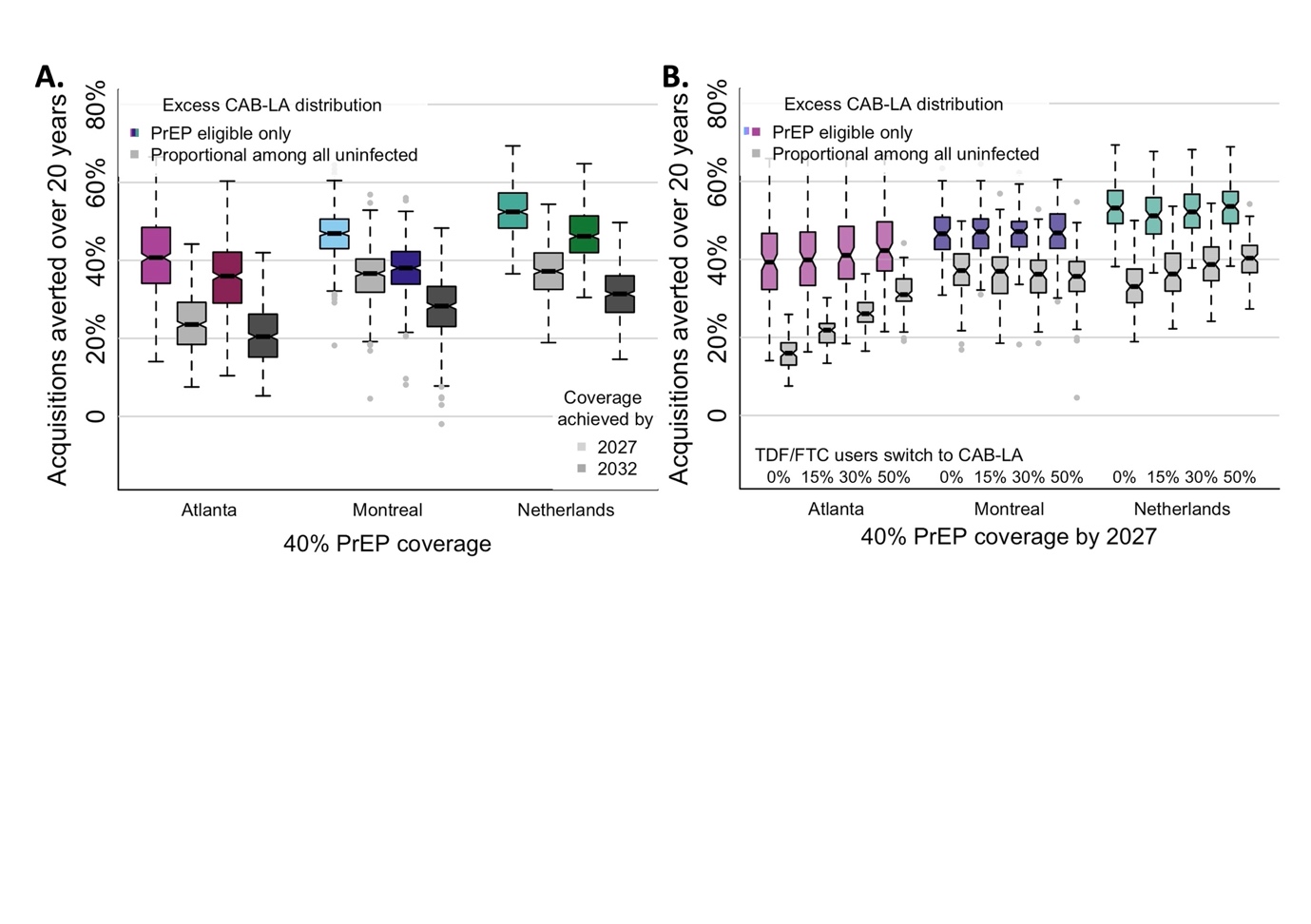
**

**Figure S9: PrEP expansion among PrEP-eligible versus proportionally among all MSM not living with HIV.** Acquisitions averted when PrEP expansion is only to those at higher risk (colors) or proportionally among all MSM not living with HIV (gray) overall (including all TDF/FTC to CAB-LA switching scenarios (A), and broken out by switching to CAB-LA (B). In Montreal, excess CAB-LA distribution was proportional by age among all MSM not living with HIV. Montreal results for excess CAB-LA distribution proportional by sexual activity among all MSM not living with HIV shown in Figure S4. *Notches in boxplot show 95% credible interval for the median.*​ *Dotted lines show maximum/minimum without outliers.*​


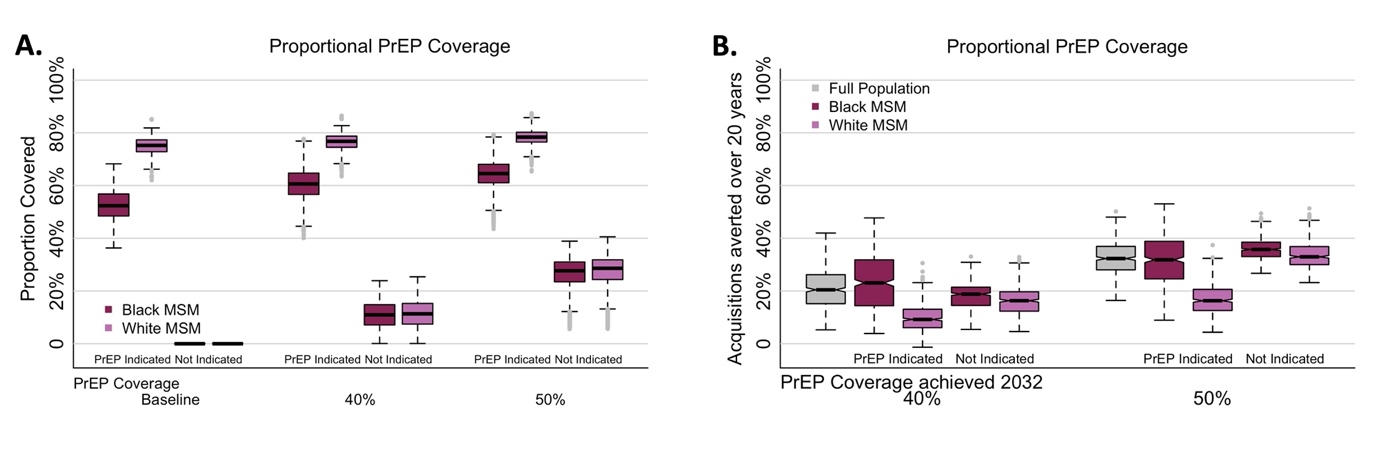


**Figure S10: PrEP expansion among PrEP-eligible versus proportionally among all MSM not living with HIV by race in the Atlanta model.** A: Proportion covered in groups of Black MSM and White MSM with and without PrEP indication. B: Acquisitions averted over 20 years in groups of Black MSM and White MSM with and without PrEP indication. *Notches in boxplot show 95% credible interval for the median.*​ *Dotted lines show maximum/minimum without outliers.*

**2. HIV Risk Coverage Calculation**

HIV risk coverage was calculated with the proportion of PrEP coverage in each PrEP eligibility/sexual partnership group at baseline and at each PrEP coverage level weighted by the proportion of new HIV acquisitions occurring in that group.

**% Risk Covered =** (% new acquisitions in the high PrEP eligibility/sexual partnership group * % of the high PrEP eligibility/sexual partnership group on PrEP) +

(% new acquisitions in the medium-high PrEP eligibility/sexual partnership group * % of the medium-high PrEP eligibility/sexual partnership group on PrEP) +

(% new acquisitions in the medium PrEP eligibility/sexual partnership group * % of the medium PrEP eligibility/sexual partnership group on PrEP) +

(% new acquisitions in the low PrEP eligibility/sexual partnership group * % of the low PrEP eligibility/sexual partnership group on PrEP)

**3. Baseline Scenarios**

**Table S1:** Baseline scenarios with initial (2022) and end (2042) metrics.

|  | **Atlanta (US)** | | **Montreal (Canada)** | | **Netherlands** | |
| --- | --- | --- | --- | --- | --- | --- |
| *Metric* | *2022* | *2042* | *2022* | *2042* | *2022* | *2042* |
| HIV prevalence  (%) | 25.63 | 18.42 | 5.60 | 1.88 | 8.54 | 6.07 |
| HIV incidence  (per 100 pys) | 2.06 | 1.62 | 0.10 | 0.03 | 0.07 | 0.001 |
| Proportion PLHIV population on ART (%) | 56.33 | 57.56 | 94.82 | 96.46 | 93.69 | 99.78 |
| Proportion PLHIV population virally suppressed (%) | 50.67 | 51.89 | 89.70 | 91.63 | *Not modeled* | *Not modeled* |
| Proportion MSM not living with HIV population on TDF/FTC (%) | 29.40 | 31.95 | 6.18 | 9.78 | 4.19 | 4.67 |
| Proportion MSM not living with HIV population PrEP-eligible (%) | 46.86 | 51.11 | 58.95 | 60.36 | 25.61 | 25.61 |

ART=antiretroviral treatment; PLHIV=people living with HIV; PrEP=pre-exposure prophylaxis.

**4. Expanded Model Descriptions**

**A. The Atlanta Model**

For Atlanta, a deterministic compartmental model of HIV transmission among sexually active MSM was used [1], stratified by age (18-24, 25+ years old), race (White, Black), and sexual activity (based on PrEP indication from the US 2017 PrEP guidelines [2]), assuming assortative mixing by age, race and sexual activity group. The epidemic was seeded in 1979 and the model was calibrated to population size, age and race distribution, proportion with a PrEP indication, HIV prevalence, proportion of people living with HIV (PLHIV) diagnosed, proportion in care, proportion virally suppressed, and proportion taking PrEP. Latin Hypercube sampling (LHS) was used to calibrate the model. Data informing the size and sexual behavior of the PrEP eligibility groups was obtained from US Centers for Disease Control (CDC) National HIV Behavioral Surveillance (NHBS) surveys, classifying MSM in the surveys as indicated or not indicated for PrEP based on 2017 US Public Health Service recommendations for MSM [2]. The recommended indications were approximated in the NHBS data analysis as reporting a main male partner living with HIV in the past twelve months OR ≥2 male partners AND either 1) condomless anal sex or 2) a bacterial sexually transmitted infection (STI) in the past 12 months [3].

Those living with HIV are distinguished by whether they have acute or chronic infection, and those with chronic infection are further stratified by set-point viral load (Log_10_ SPVL <4.0, 4.0-4.5, 4.5-5.0, >5.0) and CD4 count (CD4>500, 350-500, 200-350, <200 cells per µL). A number of different mutually exclusive HIV testing and care states are also modelled: never testing, testing (undiagnosed), on oral TDF/FTC pre-exposure prophylaxis (PrEP), on long-acting injectable CAB PrEP, or (for those living with HIV) diagnosed but not linked to care, linked into HIV care, on antiretroviral treatment (ART) and partially suppressed, on ART adherent and fully suppressed, on ART non-adherent and not suppressed, stopped taking ART.

All MSM entering the modelled population are assumed to be uninfected with HIV. MSM acquire HIV at a rate which varies by age, race, risk and PrEP use, which is related to number and type of partners (regular, casual or commercial), number of anal sex acts, condom use, circumcision status, and HIV prevalence, stage and viral suppression among partners.

In the model, those who are living with HIV and not virally suppressed transition into progressively lower CD4 count categories over time, with more rapid progression among those with higher set-point viral load. Higher levels of infectiousness are modelled for those with acute HIV acquisition and those with CD4<200 cells/µL. Changes in CD4 count are not explicitly modelled for those who are adherent to ART and virally suppressed, instead, their survival is modelled as a function of their CD4 count at ART initiation. Those who are fully suppressed are assumed to have no risk of transmitting HIV to their sexual partners, and to have reduced HIV-related mortality.

***Model parameterization***

The values and ranges used for all model parameters are given in table S2. Many of the demographic and behavioral parameters were drawn from the CDC’s National HIV Behavioral Surveillance (NHBS) cross-sectional surveys of MSM in Atlanta from 2004-2014, otherwise these came from other US studies or surveillance data. HIV progression rates, transmission probabilities and intervention efficacies were all obtained from published studies.

The percentage of MSM who were high risk, i.e. met the criteria for PrEP indication, was based on NHBS 2011 and 2014 data. The US Public Health Service suggested PrEP was indicated for MSM who: had any male sex partners in the past 6 months, were not in a monogamous partnership with a recently tested, HIV-uninfected man, and had either a) had any anal sex without condoms in the past 6 months or b) had a bacterial STI diagnosed or reported in the past 6 months. This was approximated using data that was collected in the NHBS surveys as those who: reported 1) ≥2 male partners AND either condomless anal sex or a bacterial STD in the past 12 months or 2) a main male partner living with HIV in the past 12 months.

Sexual mixing by age, race and risk were assumed to occur independently of each other, with assortative mixing by age and race based upon data from NHBS surveys, and mixing by risk assumed to vary between proportionate (random) and assortative (like-with-like) in the absence of specific data for this population. The number of sex acts per partnership were estimated from previous studies of MSM in the US.

Rates of acceptance, adherence and dropout for oral TDF/FTC PrEP were estimated by age and race from MSM participating in the US PrEP Demo project. Until 2022, PrEP was assumed to only be offered to MSM with a PrEP indication, with PrEP offer rates increasing over time from 2012 up to 2021.

**B. The Montreal Model**

For Montreal, a stochastic agent-based model of sexual HIV transmission among MSM aged ≥15 years of age was used [4]. The model stratified sexual activity behavior in three categories (≤5, 6-10, ≥11 anal sex partners per year) to parameterize intervention uptake and mixing. There was assortative mixing by age, HIV status, and sexual positioning (i.e., insertive, versatile, receptive). The epidemic was seeded in 1975 and the model was calibrated to the distribution of anal sex partner frequency, proportion and duration of regular partnerships, HIV prevalence, proportion of PLHIV diagnosed, CD4 cell count at HIV diagnosis, antiretroviral therapy (ART) coverage, proportion ever taking PrEP, and proportion of MSM not living with HIV currently taking PrEP. An Approximate Bayesian Computation Sequential Monte Carlo algorithm was used to calibrate the model. PrEP eligibility was based on the provincial PrEP guidelines [5,6] and assessed as any anal sex unprotected by condoms in the past six months and either: 1) two or more partnerships in the past six months or 2) repeat use of post-exposure prophylaxis (PEP, twice or more in their lifetime).

The model of sexual HIV transmission among Montreal MSM simulates HIV transmission and progression, partnership, and prevention use dynamics over time. Data from local epidemiological studies of Montreal MSM and the provincial surveillance system were used to parameterize and calibrate the model. Demographic processes (i.e. importation, aging, and natural death) were simulated on an annual basis at the end of the year. Based on information from four studies that occurred over 2005 – 2019, the model reproduced trends in condom use, HIV testing, ART, post-exposure prophylaxis (PEP), and PrEP in the model.

Following the Québec PrEP guidelines, oral PrEP was introduced in 2013, with HIV-negative men being considered eligible for PrEP in our model if they had any anal sex unprotected by condoms in the past six months and either: 1) two or more partnerships in the past six months or 2) repeat use of PEP (twice or more in their lifetime). An oral PrEP efficacy of 86% was assumed [7]. Men taking oral PrEP are tested for HIV following the first month of use and every subsequent three months. Beginning in 2022, CAB-LA can also be initiated by new PrEP users, with injections occurring at a time interval of four weeks between the first two doses, and eight weeks thereafter. Men taking CAB-LA additionally undergo HIV testing at each injection. Upon discontinuation of CAB-LA, protection from HIV acquisition is maintained for 10 weeks after the last dose received.

The impact of the COVID-19 pandemic on partnership and prevention dynamics was implemented in the model between March 2020 – July 2021. Briefly, men decreased their partner change rate between March – June 2020, and PEP, PrEP, ART (proxied by VLS), and HIV testing were decreased between March 2020 – July 2021, after which they returned to their pre-pandemic levels. Outside of these disruptions, the 2019 prevention uptake levels were kept constant until the end of the simulations, with the exception of PrEP which was increased as defined, starting in January, 2022. To obtain our results, each of the 100 posterior parameters sets were simulated 10 times with different seeds. The results from each simulation were averaged to provide 50 data points. The 90% CrI is reported.

**C. The Netherlands Model**

The Netherlands model was a deterministic compartmental model of HIV transmission among MSM [8,9]. The model was seeded in 1981 and calibrated to the number of PrEP users [10], estimated number of MSM diagnosed with HIV, estimated number of MSM living with HIV, yearly number of new HIV diagnoses including the proportion diagnosed in a late stage (CD4<350 cells/mm^3) and advanced stage of infection (CD4<200 cells/mm^3), and the proportion of HIV diagnosed MSM receiving ART [11]. It was stratified into four sexual activity groups (interquartile range (IQR) <1, 2-4, 8-12, 35-55 new partners every 2 years). There was assortative mixing by sexual activity group, which allowed the modeling team to identify which sexual risk group combinations resulted in the appropriately calibrated HIV epidemic [9]. LHS was used to calibrate the model. PrEP-eligible MSM included members of the two highest sexual activity groups, who together were estimated to be at substantially higher risk of acquiring HIV [9]. The model stratifies disease progression into the acute stage, three chronic stages (CD4+ T-cell count>500 cells/μL, CD4+ T-cell count 350–500 cells/μL, and CD4+ T-cell count 200–349 cells/μL) and one AIDS stage (CD4+ T-cell count<200 cells/μL).

**D. PrEP Expansion Scenarios**

When expansion occurred in PrEP-eligible groups in the Atlanta and Netherlands models, if the eligible group was large enough to achieve the target PrEP coverage, only those in the eligible group received PrEP. If the PrEP-eligible group was not large enough to achieve the targeted coverage, 90%-100% of the eligible group received PrEP and the remaining PrEP coverage target was filled from the general MSM population. When PrEP expansion occurred proportionally across the population, equal rates of PrEP initiation were assumed for all groups in the population.

***References***

1. Mitchell KM, Boily M-C, Hanscom B, Moore M, Todd J, Paz-Bailey G, et al. Estimating the impact of HIV PrEP regimens containing long-acting injectable cabotegravir or daily oral tenofovir disoproxil fumarate/emtricitabine among men who have sex with men in the United States: a mathematical modelling study for HPTN 083. . Lancet Reg Health Am. 2023;18:100416.

2. Centers for Disease Control and Prevention: US Public Health Service. Preexposure prophylaxis for the prevention of HIV infection in the United States—2017 Update: a clinical practice guideline. 2018.

3. Hoots BE, Finlayson T, Nerlander L, Paz-Bailey G, Group ftNHBSS, Wortley P, et al. Willingness to Take, Use of, and Indications for Pre-exposure Prophylaxis Among Men Who Have Sex With Men—20 US Cities, 2014. Clinical Infectious Diseases. 2016;63(5):672-7.

4. Milwid RM, Xia Y, Doyle CM, Cox J, Lambert G, Thomas R, et al. Past dynamics of HIV transmission among men who have sex with men in Montréal, Canada: a mathematical modeling study. BMC Infect Dis. 2022;22(1):233.

5. Ministère de la Santé et des Services sociaux. La prophylaxie préexposition au virus de l’immunodéficience humaine: Guide pour les professionnels de la santé du Québec. Gouvernement du Québec; 2017.

6. Ministère de la Santé et des Services sociaux. La prophylaxie préexposition au virus de l’immunodéficience humaine: Guide pour les professionnels de la santé du Québec. Gouvernement du Québec; 2019.

7. Molina JM, Capitant C, Spire B, Pialoux G, Cotte L, Charreau I, et al. On-Demand Preexposure Prophylaxis in Men at High Risk for HIV-1 Infection. N Engl J Med. 2015;373(23):2237-46.

8. Nichols BE, Boucher CAB, van der Valk M, Rijnders BJA, van de Vijver D. Cost-effectiveness analysis of pre-exposure prophylaxis for HIV-1 prevention in the Netherlands: a mathematical modelling study. Lancet Infect Dis. 2016;16(12):1423-9.

9. van de Vijver D, Richter AK, Boucher CAB, Gunsenheimer-Bartmeyer B, Kollan C, Nichols BE, et al. Cost-effectiveness and budget effect of pre-exposure prophylaxis for HIV-1 prevention in Germany from 2018 to 2058. Euro Surveill. 2019;24(7).

10. RIVM. Aantal PrEP gebruikers per GGD binnen regeling PrEP: Overzicht per eind februari 2023. 2023.

11. van Sighem AI WF, Boyd A, Smit C, Matser A, Reiss P. Monitoring Report 2022. Human Immunodeficiency Virus (HIV) Infection in the Netherlands. Amsterdam: Stichting HIV Monitoring, 2022. 2022.

**5.** **Expanded Model Parameter Tables**

**Table S2: Parameters used in the Atlanta MSM HIV transmission model, with source and justification.** *From: Mitchell KM, Boily MC, Hanscom B, Moore M, Todd J, Paz-Bailey G, Wejnert C, Liu A, Donnell DJ, Grinsztejn B, Landovitz RJ, Dimitrov DT. Estimating the impact of HIV PrEP regimens containing long-acting injectable cabotegravir or daily oral tenofovir disoproxil fumarate/emtricitabine among men who have sex with men in the United States: a mathematical modelling study for HPTN 083. Lancet Reg Health Am. 2023 Jan 17;18:100416. doi: 10.1016/j.lana.2022.100416. PMID: 36844011; PMCID: PMC9950652. (Supplementary Materials).*

| **Symbol** | **Parameter** | **Range of values^a^** | **Source/justification** |
| --- | --- | --- | --- |
| **INITIAL CONDITIONS** | | | |
| $N_{0}$ | Initial size of MSM population (1979) | 21500-30000 | 740,030 men aged 18+ in Fulton county in the 1980 census; Lower bound: Purcell et al. 2012[1] estimate % of US men had same-sex behaviour last 12 months 2.9%. Upper bound: 4%; Grey et al 2016[2] estimate 5.4% of men in Atlanta had sex with another man in the last 5 years, adjusted for ratio for last year: last 5 years from Purcell et al. |
|  | Percentage of MSM who are Black in 1979 | 10-23 | Upper bound: overall Atlanta population 1980 census. Lower bound used to improve fitting to race data in later years. |
|  | Percentage of Black MSM aged 18-24 in 1979 | 22-32 | 1990 census estimate (27%) ±5 percentage points (pp) |
|  | Percentage of White MSM aged 18-24 in 1979 | 6-16 | 1990 census estimate (11%) ±5 percentage points (pp) |
| $m_{0,0,high}$ | Percentage of Black 18-24 year old MSM meeting criteria for a PrEP indication in 1979 and among new incoming MSM | 14-76 | NHBS data 2011-2014 (highest and lowest from 95% CI); PrEP indication if reported 1) ≥2 male partners AND (condomless anal sex OR bacterial STD) in the past 12 months or 2) main male partner living with HIV in the past 12 months |
| $m_{0,1,high}$ | Percentage of White 18-24 year old MSM meeting criteria for a PrEP indication in 1979 and among new incoming MSM | 40-61 | NHBS data 2011-2014 (highest and lowest from 95% CI); PrEP indication if reported 1) ≥2 male partners AND (condomless anal sex OR bacterial STD) in the past 12 months or 2) main male partner living with HIV in the past 12 months |
| $m_{1,0,high}$ | Percentage of Black 25+ year old MSM meeting criteria for a PrEP indication in 1979 and among new incoming MSM | 13-67 | NHBS data 2011-2014 (highest and lowest from 95% CI); PrEP indication if reported 1) ≥2 male partners AND (condomless anal sex OR bacterial STD) in the past 12 months or 2) main male partner living with HIV in the past 12 months |
| $m_{1,1,high}$ | Percentage of Black 25+ year old MSM meeting criteria for a PrEP indication in 1979 and among new incoming MSM | 37-61 | NHBS data 2011-2014 (highest and lowest from 95% CI); PrEP indication if reported 1) ≥2 male partners AND (condomless anal sex OR bacterial STD) in the past 12 months or 2) main male partner living with HIV in the past 12 months |
|  | HIV prevalence Black MSM 1979 (%) | 0.1-2 | Assumption |
|  | HIV prevalence White MSM 1979 (%) | 0.1-1 | Assumption |
| ***Demography*** | | | |
| $\Gamma$ | Rate at which new MSM join the sexually active MSM population (number per year) | 3000-5500 | estimate – fitting to NHBS demography |
| $m_{black}$ | Percentage of new incoming MSM who are Black, 2014^b^ | 45-75 | Lower bound: % of current MSM who are Black NHBS 2011; Upper bound set to fit data on race (% of current MSM who are Black NHBS 2014: 62%) |
| $m_{black}\_inc$ | Annual increase in percentage of new incoming MSM who are Black, 1979-2014 | 0.2-1.5 | Estimate – fitting to NHBS demography |
| $m_{young,0}$ | Percentage of new incoming Black MSM who are aged 18-24 years old | 51-70 | % of Black MSM in NHBS who say they entered sexually active Atlanta MSM population aged <25 – 2014 NHBS |
| $m_{young,1}$ | Percentage of new incoming White MSM who are aged 18-24 years old | 30-56 | % of White MSM in NHBS who say they entered sexually active Atlanta MSM population aged <25 – 2014 NHBS; lower bound reduced to fit data on age |
| $\pi_{0}$ | rate of moving from 18-24 year old age group to >24 year old age group, Black MSM, per year | 0.17 (fixed) | Mean age at joining the local MSM population in NHBS 2011 for 18-24 yr old MSM ~16 yrs old (95% CI 15-17), in NHBS 2014 17.5 yrs old (17-18) |
| $\pi_{1}$ | rate of moving from 18-24 year old age group to >24 year old age group, White MSM, per year | 0.17-0.25 | Mean age at joining the local MSM population in NHBS 2011 for 18-24 yr old MSM ~19 yrs old (95% CI 18-20), in NHBS 2014 ~19.5 yrs old (18-21). Assume they spend 4-6 years in 18-24 yr old age group. |
| $\mu_{0,0}$ | Non-HIV related death rate, 18-24 year old Black men, per year | 0.0014-0.0018 | CDC WONDER database data for Georgia[3]; data for 15-24 years olds |
| $\mu_{1,0}$ | Non-HIV related death/leaving rate, >24 year old Black men, per year | 0.037-0.11 | CDC WONDER database data for Georgia[3]: average death rate over ages 25-64 years old, add on 1/29 (double current duration as an MSM) to upper bound, additionally assume extra rate of  ceasing to attend NHBS venues |
| $\mu_{0,1}$ | Non-HIV related death rate, 18-24 year old White men, per year | 0.0011-0.0015 | CDC WONDER database data for Georgia[3]; data for 15-24 years olds |
| $\mu_{1,1}$ | Non-HIV related death/leaving rate, >24 year old White men, per year | 0.035-0.1 | CDC WONDER database data for Georgia[3]: average death rate over ages 25-64 years old, add on 1/43 (double current duration as an MSM) to upper bound, additionally assume extra rate of  ceasing to attend NHBS venues |
| ***Sexual behaviour*** | | | |
| $n_{1}$ | Number of anal sex acts per main partnership | 40-470 | 48.2-85.1 sex episodes/year with main partners [4], partnerships last 3.5-5.5 years [5,6], but assume some are shorter (~1 year) |
| $n_{2}$ | Number of anal sex acts per casual partnership | 1-40 | In line with previous fitted estimates for MSM in Baltimore[7] |
| $n_{3}$ | Number of anal sex acts per commercial partnership | 1-40 | In line with previous fitted estimates for MSM in Baltimore[7] |
| $c_{0,0,0,1}$ | Number of new main partners per year, 18-24 year old Black MSM without a PrEP indication | 0.29-0.65 | NHBS 2014 - number of main male anal sex partners in the last 12 months multiplied by the proportion of all main partners in the past 12 months who were new partners. |
| $c_{0,0,0,2}$ | Number of new casual partners per year, 18-24 year old Black MSM without a PrEP indication 2011 onwards^b^ | 0.4-1.45 | NHBS 2014 - number of casual male anal sex partners in the last 12 months multiplied by the proportion of all casual partners in the past 12 months who were new partners, subtracting the number of new commercial partners |
| $c_{0,0,0,3}$ | Number of new commercial partners per year, 18-24 year old Black MSM without a PrEP indication 2011 onwards^b^ | 0-0.06 | NHBS 2014 - number of exchange male sex partners in the last 12 months multiplied by the proportion of all casual partners in the past 12 months who were new partners and multiplied by the proportion of casual partners with whom they had anal sex |
| $c_{0,0,1,1}$ | Number of new main partners per year, 18-24 year old Black MSM with a PrEP indication | 0.65-1.12 | NHBS 2014 - number of main male anal sex partners in the last 12 months multiplied by the proportion of all main partners in the past 12 months who were new partners. |
| $c_{0,0,1,2}$ | Number of new casual partners per year, 18-24 year old Black MSM with a PrEP indication 2011 onwards^b^ | 1.5-2.93 | NHBS 2014 - number of casual male anal sex partners in the last 12 months multiplied by the proportion of all casual partners in the past 12 months who were new partners, subtracting the number of new commercial partners |
| $c_{0,0,1,3}$ | Number of new commercial partners per year, 18-24 year old Black MSM with a PrEP indication 2011 onwards^b^ | 0.0005-0.45 | NHBS 2014 - number of exchange male sex partners in the last 12 months multiplied by the proportion of all casual partners in the past 12 months who were new partners and multiplied by the proportion of casual partners with whom they had anal sex |
| $c_{1,0,0,1}$ | Number of new main partners per year, >24 year old Black MSM without a PrEP indication | 0.41-0.66 | NHBS 2014 - number of main male anal sex partners in the last 12 months multiplied by the proportion of all main partners in the past 12 months who were new partners. |
| $c_{1,0,0,2}$ | Number of new casual partners per year, >24 year old Black MSM without a PrEP indication 2011 onwards^b^ | 0.52-0.93 | NHBS 2014 - number of casual male anal sex partners in the last 12 months multiplied by the proportion of all casual partners in the past 12 months who were new partners, subtracting the number of new commercial partners |
| $c_{1,0,0,3}$ | Number of new commercial partners per year, >24 year old Black MSM without a PrEP indication 2011 onwards^b^ | 0.01-0.18 | NHBS 2014 - number of exchange male sex partners in the last 12 months multiplied by the proportion of all casual partners in the past 12 months who were new partners and multiplied by the proportion of casual partners with whom they had anal sex |
| $c_{1,0,1,1}$ | Number of new main partners per year, >24 year old Black MSM with a PrEP indication | 0.41-0.62 | NHBS 2014 - number of main male anal sex partners in the last 12 months multiplied by the proportion of all main partners in the past 12 months who were new partners. |
| $c_{1,0,1,2}$ | Number of new casual partners per year, >24 year old Black MSM with a PrEP indication 2011 onwards^b^ | 2.78-5.14 | NHBS 2014 - number of casual male anal sex partners in the last 12 months multiplied by the proportion of all casual partners in the past 12 months who were new partners, subtracting the number of new commercial partners |
| $c_{1,0,1,3}$ | Number of new commercial partners per year, >24 year old Black MSM with a PrEP indication 2011 onwards^b^ | 0.19-1.67 | NHBS 2014 - number of exchange male sex partners in the last 12 months multiplied by the proportion of all casual partners in the past 12 months who were new partners and multiplied by the proportion of casual partners with whom they had anal sex |
| $c_{0,1,0,1}$ | Number of new main partners per year, 18-24 year old White MSM without a PrEP indication | 0.7-2.24 | NHBS 2014 - number of main male anal sex partners in the last 12 months multiplied by the proportion of all main partners in the past 12 months who were new partners. |
| $c_{0,1,0,2}$ | Number of new casual partners per year, 18-24 year old White MSM without a PrEP indication 2011 onwards^b^ | 0-1.38 | NHBS 2014 - number of casual male anal sex partners in the last 12 months multiplied by the proportion of all casual partners in the past 12 months who were new partners, subtracting the number of new commercial partners |
| $c_{0,1,0,3}$ | Number of new commercial partners per year, 18-24 year old White MSM without a PrEP indication 2011 onwards^b^ | 0-0.07 | NHBS 2014 - number of exchange male sex partners in the last 12 months multiplied by the proportion of all casual partners in the past 12 months who were new partners and multiplied by the proportion of casual partners with whom they had anal sex |
| $c_{0,1,1,1}$ | Number of new main partners per year, 18-24 year old White MSM with a PrEP indication | 0.18-0.62 | NHBS 2014 - number of main male anal sex partners in the last 12 months multiplied by the proportion of all main partners in the past 12 months who were new partners. |
| $c_{0,1,1,2}$ | Number of new casual partners per year, 18-24 year old White MSM with a PrEP indication 2011 onwards^b^ | 0-1.45 | NHBS 2014 - number of casual male anal sex partners in the last 12 months multiplied by the proportion of all casual partners in the past 12 months who were new partners, subtracting the number of new commercial partners |
| $c_{0,1,1,3}$ | Number of new commercial partners per year, 18-24 year old White MSM with a PrEP indication 2011 onwards^b^ | 0-0.19 | NHBS 2014 - number of exchange male sex partners in the last 12 months multiplied by the proportion of all casual partners in the past 12 months who were new partners and multiplied by the proportion of casual partners with whom they had anal sex |
| $c_{1,1,0,1}$ | Number of new main partners per year, >24 year old White MSM without a PrEP indication | 0.2-0.43 | NHBS 2014 - number of main male anal sex partners in the last 12 months multiplied by the proportion of all main partners in the past 12 months who were new partners. |
| $c_{1,1,0,2}$ | Number of new casual partners per year, >24 year old White MSM without a PrEP indication 2011 onwards^b^ | 0.78-2.92 | NHBS 2014 - number of casual male anal sex partners in the last 12 months multiplied by the proportion of all casual partners in the past 12 months who were new partners, subtracting the number of new commercial partners |
| $c_{1,1,0,3}$ | Number of new commercial partners per year, >24 year old White MSM without a PrEP indication 2011 onwards^b^ | 0-0.06 | NHBS 2014 - number of exchange male sex partners in the last 12 months multiplied by the proportion of all casual partners in the past 12 months who were new partners and multiplied by the proportion of casual partners with whom they had anal sex |
| $c_{1,1,1,1}$ | Number of new main partners per year, >24 year old White MSM with a PrEP indication | 0.21-0.44 | NHBS 2014 - number of main male anal sex partners in the last 12 months multiplied by the proportion of all main partners in the past 12 months who were new partners. |
| $c_{1,1,1,2}$ | Number of new casual partners per year, >24 year old White MSM with a PrEP indication 2011 onwards^b^ | 3.69-16.4 | NHBS 2014 - number of casual male anal sex partners in the last 12 months multiplied by the proportion of all casual partners in the past 12 months who were new partners, subtracting the number of new commercial partners |
| $c_{1,1,1,3}$ | Number of new commercial partners per year, >24 year old White MSM with a PrEP indication 2011 onwards^b^ | 0-2.52 | NHBS 2014 - number of exchange male sex partners in the last 12 months multiplied by the proportion of all casual partners in the past 12 months who were new partners and multiplied by the proportion of casual partners with whom they had anal sex |
| Partner_number_decline_low | absolute decline per year in the number of new casual or commercial partners, for MSM without a PrEP indication | 0.51-0.71 | From trends in NHBS data on number of commercial and causal partners 2008-2014 for MSM without a PrEP indication, and trends in number of commercial and casual partners 2004-2014 for all MSM |
| Partner_number_decline_high | absolute decline per year in the number of new casual or commercial partners, for MSM with a PrEP indication | 0.92-1.12 | From trends in NHBS data on number of commercial and causal partners 2008-2014 for MSM with a PrEP indication, and trends in number of commercial and casual partners 2004-2014 for all MSM |
| $\varepsilon_{a}$ | Mixing parameter for mixing by age scaled between fully proportionate (0) and fully assortative (1) | 0.32-0.62 | 0.53 estimated from NHBS 2011 data on last partner, and 0.41 from 2014 data on last partner; range takes into account uncertainty due to incomplete data on age of last partner |
| $\varepsilon_{r}$ | Mixing parameter for mixing by race scaled between fully proportionate (0) and fully assortative (1) | 0.69-0.79 | 0.75 estimated from NHBS 2011 data on last partner and 0.73 from NHBS 2014 data on last partner |
| $\varepsilon_{p}$ | Mixing parameter for mixing by PrEP indication scaled between fully proportionate (0) and fully assortative (1) | 0-1 | No data; full range explored |
| $s_{c,1,0,0}$ | Percentage of sex acts in which a condom is used, main partnerships where both partners are Black and one or both partners do not have a PrEP indication, 2014 onwards^b^ | 41-69 | condom use last sex act reported by Black MSM without a PrEP indication with main partners NHBS 2014 |
| $s_{c,1,1,0}$ | Percentage of sex acts in which a condom is used, main partnerships where one or both partners are White and one or both partners do not have a PrEP indication, 2014 onwards^b^ | 21-57 | condom use last sex act reported by White MSM without a PrEP indication with main partners NHBS 2014 |
| $s_{c,2,0,0}{,s}_{c,2,1,0}$ | Percentage of sex acts in which a condom is used, casual partnerships (any race partner) where one or both partners do not have a PrEP indication, 2014 onwards^b^ | 40-91 | condom use last sex act reported in casual partnerships by MSM without a PrEP indication NHBS 2014 – range from values for Black and White MSM |
| $s_{c,3,0,0},s_{c,3,1,0}$ | Percentage of sex acts in which a condom is used, commercial partnerships (any race partner) where one or both partners do not have a PrEP indication, 2014 onwards^b^ | 40-91 | Assumed to be the same as casual |
| $s_{c,1,0,1}$ | Percentage of sex acts in which a condom is used, main partnerships where both partners are Black and both partners have a PrEP indication, 2014 onwards^b^ | 18-56 | condom use last sex act reported by Black MSM with a PrEP indication with main partners NHBS 2014 |
| $s_{c,1,1,1}$ | Percentage of sex acts in which a condom is used, main partnerships where one or both partners are White and both partners have a PrEP indication, 2014 onwards^b^ | 3-31 | condom use last sex act reported by White MSM with a PrEP indication with main partners NHBS 2014 |
| $s_{c,2,0,1}{,s}_{c,2,1,1}$ | Percentage of sex acts in which a condom is used, casual partnerships (any race partner) where both partners have a PrEP indication, 2014 onwards^b^ | 17-60 | condom use last sex act reported in casual partnerships by MSM with a PrEP indication NHBS 2014 – range from values for Black and White MSM |
| $s_{c,3,0,1},s_{c,3,1,1}$ | Percentage of sex acts in which a condom is used, commercial partnerships (any race partner) where both partners have a PrEP indication, 2014 onwards^b^ | 17-60 | Assumed to be the same as casual |
| Condom_change_1 | Yearly change in % of sex acts in which condoms are used, all partnerships 1996-2008 | -0.94 to -0.31 | From study by Kalichman et al[8], decline between 1997 and 2005, ±50% |
| Condom_change_2 | Yearly change in % of sex acts in which condoms are used, all partnerships between 2008 and 2014 | -4.5 to -0.1 | From trend in condom use at last sex act NHBS 2011-2014, taking into account variation across race and PrEP-indication groups, and across main and casual partners. |
| ***HIV disease progression*** | |  |  |
| $1/\gamma_{a}$ | Average duration of acute infection, months | 2-6 | Systematic review and analysis of data from Uganda[9,10] |
| $\alpha_{0,0,z}$ | HIV-related death rate for those with acute HIV infection, per year | 0 (fixed) | assumption |
| $\alpha_{1,y,0}$  $\alpha_{1,y,1}$  $\alpha_{1,y,2}$  $\alpha_{1,y,3}$  $\alpha_{1,y,4}$  $\alpha_{1,y,9}$  $\alpha_{1,y,10}$ | HIV-related death rate for those with CD4>500, off ART, per year | 0.0009-0.0054 | aged 25-44 in the European CASCADE cohort [11]; general population death rate subtracted |
| $\alpha_{2,y,0}$  $\alpha_{2,y,1}$  $\alpha_{2,y,2}$  $\alpha_{2,y,3}$  $\alpha_{2,y,4}$  $\alpha_{2,y,9}$  $\alpha_{2,y,10}$ | HIV-related death rate for those with CD4 350-500, off ART, per year | 0.0009-0.0069 | aged 25-44 in the European CASCADE cohort [11]; general population death rate subtracted |
| $\alpha_{3,y,0}$  $\alpha_{3,y,1}$  $\alpha_{3,y,2}$  $\alpha_{3,y,3}$  $\alpha_{3,y,4}$  $\alpha_{3,y,9}$  $\alpha_{3,y,10}$ | HIV related death rate for those with CD4 200-350, off ART, per year | 0.0045-0.0135 | aged 25-44 in the European CASCADE cohort [11]; general population death rate subtracted |
| 1/$\alpha_{4,1,1}$ | Inverse of HIV-related death rate for those with CD4<200, SPVL<4.0, off ART (years) | 3.28-12.87 | Netherlands ATHENA cohort [12] |
| 1/$\alpha_{4,2,1}$ | Inverse of HIV-related death rate for those with CD4<200, SPVL 4.0-4.5, off ART (years) | 1.43-6.09 | Netherlands ATHENA cohort [12] |
| 1/$\alpha_{4,3,1}$ | Inverse of HIV-related death rate for those with CD4<200, SPVL 4,5-5,0, off ART (years) | 4.41-23.64 | Netherlands ATHENA cohort [12] |
| 1/$\alpha_{4,4,1}$ | Inverse of HIV-related death rate for those with CD4<200, SPVL>5.0, off ART (years) | 1.32-3.59 | Netherlands ATHENA cohort [12] |
| $\alpha_{1,y,5}$,$\alpha_{2,y,5}$  $\alpha_{1,y,6}$,$\alpha_{2,y,6}$  $\alpha_{1,y,7}$,$\alpha_{2,y,7}$  $\alpha_{1,y,8}$,$\alpha_{2,y,8}$ | HIV-related mortality for those with CD4>500 or CD4 350-500 at start of treatment, for 1^st^ , 2^nd^ and subsequent years on ART, per year | 0-0.003 | European and North American cohorts; from probabilities for those with CD4>350 [13]; general population death rate subtracted [14] |
| $a_{1}$ | Relative mortality of those with CD4 200-350 vs CD4>350 at start of treatment, 1^st^ year on ART | 1.2-2.8 | European and North American cohorts[15] |
| $a_{2}$ | Relative mortality of those with CD4 200-350 vs CD4>350 at start of treatment, 2^nd^ year on ART | 1-2.2 | European and North American cohorts [15] Upper limit reduced to give main estimate as midpoint |
| $a_{3}$ | Relative mortality of those with CD4 200-350 vs CD4>350 at start of treatment, 3^rd^ year + on ART | 1-1.4 | European and North American cohorts [15] Upper limit reduced to give main estimate as midpoint |
| $a_{4}$ | Relative mortality of those with CD4 <200 vs CD4>350 at start of treatment, 1^st^ year on ART | 1.8-5.2 | European and North American cohorts [15] Main estimate and lower bound: CD4 100-199; upper bound from those with CD4 25-49 |
| $a_{5}$ | Relative mortality of those with CD4 <200 vs CD4>350 at start of treatment, 2^nd^ year on ART | 1.3-6.2 | European and North American cohorts [15] Main estimate and lower bound: CD4 100-199; upper bound from those with CD4 25-49 |
| $a_{6}$ | Relative mortality of those with CD4 <200 vs CD4>350 at start of treatment, 3^rd^ year + on ART | 1-3.2 | European and North American cohorts [15] Main estimate and lower bound: CD4 100-199; upper bound from those with CD4 50-99 |
| $b_{1}$ | Relative mortality of those with AIDS before ART initiation vs without, 1^st^ year on ART | 3.0-4.8 | European and North American cohorts [15] |
| $b_{2},b_{3}$ | Relative mortality of those with AIDS before ART initiation vs without, 2^nd^ , 3^rd^ + years on ART | 1.4-2.6 | European and North American cohorts [15] |
| $k_{4}$ | Percentage of those starting ART with CD4<200 who have a prior AIDS diagnosis | 40-60 | US data [16] |
| $\theta_{2}$ | Percentage of HIV-positive MSM with a SPVL 4.0-4.5 | 25 (fixed) | Netherlands ATHENA cohort [12]; US MSM (MACS cohort)[17,18] |
| $\theta_{3}$ | Percentage of HIV-positive MSM with a SPVL 4.5-5.0 | 25-40 | Netherlands ATHENA cohort [12]; US MSM (MACS cohort)[17,18] |
| $\theta_{4}$ | Percentage of HIV-positive MSM with a SPVL >5.0 | 10-25 | Netherlands ATHENA cohort [12]; US MSM (MACS cohort)[18] |
| ${1/\gamma}_{1,1}$ | Average duration spent with CD4>500 cells per µL, for those with SPVL <4.0 (years) | 4.56-6.37 | Netherlands ATHENA cohort [12] |
| ${1/\gamma}_{2,1}$ | Average duration spent with CD4 350-500, for those with SPVL <4.0 (years) | 2.98-4.53 | Netherlands ATHENA cohort [12] |
| ${1/\gamma}_{3,1}$ | Average duration spent with CD4 200-350, for those with SPVL <4.0 (years) | 5.04-13.69 | Netherlands ATHENA cohort [12] |
| ${1/\gamma}_{1,2}$ | Average duration spent with CD4>500, for those with SPVL 4.0-4.5 (years) | 2.68-3.64 | Netherlands ATHENA cohort [12] |
| ${1/\gamma}_{2,2}$ | Average duration spent with CD4 350-500, for those with SPVL 4.0-4.5 (years) | 2.65-3.64 | Netherlands ATHENA cohort [12] |
| ${1/\gamma}_{3,2}$ | Average duration spent with CD4 200-350, for those with SPVL 4.0-4.5 (years) | 5.46-15.55 | Netherlands ATHENA cohort [12] |
| ${1/\gamma}_{1,3}$ | Average duration spent with CD4>500, for those with SPVL 4.5-5.0 (years) | 2.08-2.64 | Netherlands ATHENA cohort [12] |
| ${1/\gamma}_{2,3}$ | Average duration spent with CD4 350-500, for those with SPVL 4.5-5.0 (years) | 1.98-2.72 | Netherlands ATHENA cohort [12] |
| ${1/\gamma}_{3,3}$ | Average duration spent with CD4 200-350, for those with SPVL 4.5-5.0 (years) | 4.73-10.22 | Netherlands ATHENA cohort [12] |
| ${1/\gamma}_{1,4}$ | Average duration spent with CD4>500, for those with SPVL ≥5.0 (years) | 1.28-1.76 | Netherlands ATHENA cohort [12] |
| ${1/\gamma}_{2,4}$ | Average duration spent with CD4 350-500, for those with SPVL ≥5.0 (years) | 1.22-1.69 | Netherlands ATHENA cohort [12] |
| ${1/\gamma}_{3,4}$ | Average duration spent with CD4 200-350, for those with SPVL ≥5.0 (years) | 2.12-4.19 | Netherlands ATHENA cohort [12] |
| ${1/\sigma}_{0}$ | Average duration from ART initiation to viral suppression (VL < 200 copies/ml) for those with acute HIV infection (months) | 3.93-8.50 | Pregnant women, Kenya[19] |
| ${1/\sigma}_{1}$ | Average duration from ART initiation to viral suppression (VL < 200 copies/ml) for those with log_10_ SPVL <4.0 (months) | 0.95-4.1 | Data from Johns Hopkins (Baltimore) and Fenway (Boston); estimate is weighted average of median values from 2 sites[20] |
| ${1/\sigma}_{2}$ | Average duration from ART initiation to viral suppression (VL < 200 copies/ml) for those with log_10_ SPVL 4.0-4.5 (months) | 1.03-4.75 | Data from Johns Hopkins (Baltimore) and Fenway (Boston); estimate is weighted average of median values from 2 sites[20] |
| ${1/\sigma}_{3}$ | Average duration from ART initiation to viral suppression (VL < 200 copies/ml) for those with log_10_ SPVL 4.5-5.0 (months) | 1.4-6.43 | Data from Johns Hopkins (Baltimore) and Fenway (Boston); estimate is weighted average of median values from 2 sites[20] |
| ${1/\sigma}_{4}$ | Average duration from ART initiation to viral suppression (VL < 200 copies/ml) for those with log_10_ SPVL >5.0 (months) | 2.03-6.49 | Data from Johns Hopkins (Baltimore) and Fenway (Boston); estimate is weighted average of median values from 2 sites[20] |
| $f_{1,1}$ | Percentage with CD4 >500 after seroconversion, for those with SPVL <4.0 | 81-91 | Netherlands ATHENA cohort [12] |
| $f_{3,1}$ | Percentage with CD4 200-350 after seroconversion, for those with SPVL <4.0 | 0-4 | Netherlands ATHENA cohort [12] |
| $f_{4,1}$ | Percentage with CD4 <200 after seroconversion, for those with SPVL <4.0 | 0 (fixed) | Netherlands ATHENA cohort [12] |
| $f_{1,2}$ | Percentage with CD4 >500 after seroconversion, for those with SPVL 4.0-4.5 | 72-83 | Netherlands ATHENA cohort [12] |
| $f_{3,2}$ | Percentage with CD4 200-350 after seroconversion, for those with SPVL 4.0-4.5 | 1-5 | Netherlands ATHENA cohort [12] |
| $f_{4,2}$ | Percentage with CD4 <200 after seroconversion, for those with SPVL 4.0-4.5 | 0 (fixed) | Netherlands ATHENA cohort [12] |
| $f_{1,3}$ | Percentage with CD4 >500 after seroconversion, for those with SPVL 4.5-5.0 | 69-79 | Netherlands ATHENA cohort [12] |
| $f_{3,3}$ | Percentage with CD4 200-350 after seroconversion, for those with SPVL 4.5-5.0 | 3-8 | Netherlands ATHENA cohort [12] |
| $f_{4,3}$ | Percentage with CD4 <200 after seroconversion, for those with SPVL 4.5-5.0 | 0 (fixed) | Netherlands ATHENA cohort [12] |
| $f_{1,4}$ | Percentage with CD4 >500 after seroconversion, for those with SPVL ≥5.0 | 64-77 | Netherlands ATHENA cohort [12] |
| $f_{3,4}$ | Percentage with CD4 200-350 after seroconversion, for those with SPVL ≥5.0 | 2-7 | Netherlands ATHENA cohort [12] |
| $f_{4,4}$ | Percentage with CD4 <200 after seroconversion, for those with SPVL ≥5.0 | 0 (fixed) | Netherlands ATHENA cohort [12] |
| ***Transmission probabilities*** | | | |
| $d_{1}$ | Relative infectiousness of HIV-positive partner in acute stage of infection vs chronic & CD4>200 (off ART) | 4.47-18.81 | Systematic review of studies of heterosexual transmission[10] |
| $d_{2}$ | Relative infectiousness of HIV-positive partner in late stage of infection – CD4<200 cells per µL vs chronic and CD4>200 (off ART) | 2-8 | Cohort study among African heterosexual adults[21,22] |
| $\beta$ | Average probability of acquiring HIV infection per sex act with an HIV-positive partner with chronic untreated infection | 0.0007-0.0285 | Systematic review of anal transmission and study among Australian MSM[23,24]; assume 50% of sex acts are insertive |
| $h_{1}$ | Relative infectiousness of HIV-positive person with log_10_ SPVL <4.0 vs 4.0-4.5 | 0.337-0.68 | [25] Inverse of pooled increase in transmissibility per log10 decrease in viral load |
| $h_{2}$ | Relative infectiousness of HIV-positive person with log_10_ SPVL 4.0-4.5 vs 4.0-4.5 | 1 (fixed) | _ |
| $h_{3}$ | Relative infectiousness of HIV-positive person with log_10_ SPVL 4.5-5.0 vs 4.0-4.5 | 1 (fixed) | _ |
| $h_{4}$ | Relative infectiousness of HIV-positive person with log_10_ SPVL >5.0 vs 4.0-4.5 | 1.47-2.97 | [25] pooled increase in transmissibility per log10 increase in viral load |
| $r_{0}$ | Relative susceptibility of Black MSM | 1 (fixed) |  |
| $r_{1}$ | Relative susceptibility of White MSM | 0.2-1 | Varied to fit race-specific HIV prevalence; accounts for remaining differences in risk by race not captured by other parameters |
| ***Intervention behaviour*** | | | |
| $p_{0}$ | Percentage of new Black entrants to MSM population who never routinely test for HIV | 1-11 | NHBS Atlanta MSM 2011-2014 (highest and lowest from 95% CI across both years): % of those aged >24 years old who report never testing for HIV |
| $p_{ratio}$ | Ratio of percentage of new entrants who never routinely test for HIV for White:Black MSM | 0.13-0.63 | NHBS Atlanta MSM ratio in 2014 (lower bound), ratio in 2011 (upper bound) for % of those aged >24 years old who report never testing for HIV White:Black |
| ${test}_{0,0}$ | Percentage of undiagnosed Black MSM aged 18-24 testing for HIV in the last year, 2004 onwards^b^ | 29-48 (diagnosis fitting) | NHBS data 2011-2014(highest and lowest from 95% CI), self-reported HIV negative men, reduced by 50-60% for fitting to diagnosis data; converted into rate of testing at least once per year in the model ($\tau_{0.0.1}$) |
| $test_{0,1}$ | Percentage of undiagnosed White MSM aged 18-24 testing for HIV in the last year, 2004 onwards^b^ | 13-46 (diagnosis fitting) | NHBS data 2011-2014(highest and lowest from 95% CI), self-reported HIV negative men, reduced by 50-60% for fitting to diagnosis data; converted into rate of testing at least once per year in the model ($\tau_{0.1.1}$) |
| ${test}_{1,0}$ | Percentage of undiagnosed Black MSM aged >24 years old testing for HIV in the last year, 2004 onwards^b^ | 25-42 (diagnosis fitting) | NHBS data 2011-2014(highest and lowest from 95% CI), self-reported HIV negative men, reduced by 50-60% for fitting to diagnosis data; converted into rate of testing at least once per year in the model ($\tau_{1.0.1}$) |
| ${test}_{1,1}$ | Percentage of undiagnosed White MSM aged >24 years old testing for HIV in the last year, 2004 onwards^b^ | 25-42 (diagnosis fitting) | NHBS data 2011-2014(highest and lowest from 95% CI), self-reported HIV negative men, reduced by 50-60% for fitting to diagnosis data; converted into rate of testing at least once per year in the model ($\tau_{1.1.1}$) |
| ${test}_{early}$ | Percentage of all MSM who tested for HIV in the last year, 1996 | 8-15 (diagnosis fitting) | MSM in national NHSDA survey 1996 [26], reduced by 50-60% for fitting to diagnosis data; converted into rate of testing at least once per year in the model |
| $\tau_{0.0.2}$,$\tau_{0.1.2}$,$\tau_{1.0.2}$,$\tau_{1.1.2}$ | Number of HIV tests per year among those on PrEP | 4 (fixed) | Based on recommended frequency of HIV testing for those on PrEP at least 4 times per year[27] |
| *q*_1,1_ | Percentage of White MSM not on PrEP testing positive for HIV who link to care straight away, 2008 onwards^b^ | 70-86 | Lower bound: Georgia DPH report 2012[28] – calculated from % all and % White linking to care within 3 months of diagnosis  Upper bound: Georgia DPH report 2014[29]: % White MSM linking within 1 month of diagnosis |
| *q*_2,0,_ *q*_2,1_ | Percentage of Black, White MSM on PrEP testing positive for HIV who link to care straight away, 2012 onwards | 100 | Assumption |
| $\epsilon_{1}$ | Rate of linkage to care for White MSM not linking immediately or dropped out, per year |  | Estimate |
| linkage_inc | Annual absolute increase in percentage of White MSM who link to care straight away after testing positive for HIV, 1979-2008 | 3.5 (fixed) | From changes for MSM in national CDC data [30,31] |
| $\epsilon_{ratio}$ | Ratio of rates of linkage to care for Black:White MSM (ratio also applied to percentage linking immediately after diagnosis for those not on PrEP) | 0.76-1.02 | Georgia DPH data 2011, 2014[29,32], National estimates for MSM from NHBS and NHSS data [33,34] |
| $\omega_{1}$ | Ratio of rate of dropout from care: rate of dropout from ART for White MSM | 1-7 | Estimates from US studies - risk of dropout from care for those on vs off ART [35-37] |
| $\omega_{ratio}$ | Ratio of dropout from care for White:Black MSM | 0.46-1.54 | Studies of US cohort data and US HIV surveillance data [35-40] |
| $\xi_{x,1}$ | Rate of initiation onto ART from care for White MSM, when meeting CD4 criteria^c^, per year^b^ | 1.1-2 | Assuming CD4 testing less frequent than the recommendation in national guidelines (every 3-6 months), with acceptance of 80-90% [41] |
| $\xi_{ratio}$ | Ratio of ART initiation rate for Black:White MSM | 0.4-1.0 | US ART cohort data[37] |
| ${\psi_{0,z}, \psi}_{1,z}$ | Rate of starting HAART due to AIDS symptoms, CD4>500, per year (post-1996) | 0.002-0.01 | Incidence of AIDS-defining illness among ART naives, CASCADE collaboration [42]; similar estimates from EURO-COORD data analysis [43] |
| $\psi_{2,z}$ | Rate of starting HAART due to AIDS symptoms, CD4 350-500, per year (post-1996) | 0.008-0.015 | Incidence of AIDS-defining illness among ART naives, CASCADE collaboration [42]; similar estimates from EURO-COORD data analysis [43] |
| $\psi_{3,z}$ | Rate of starting HAART due to AIDS symptoms, CD4 200-350, per year (post-1996) | 0.018-0.032 | Incidence of AIDS-defining illness among ART naives, CASCADE collaboration [42]; similar estimates from EURO-COORD data analysis [43] |
| $\psi_{4,z}$ | Rate of starting HAART due to AIDS symptoms, CD4<200, per year (post-1996) | 0.173-0.262 | Incidence of AIDS-defining illness among ART naives, CASCADE collaboration [42] |
| $\chi_{1}$ | Percentage of White MSM initiating ART who are adherent (achieve viral suppression) | 73-99 | Studies of US cohort data and US HIV surveillance data [33,44-46] |
| $\chi_{ratio}$ | Ratio of percentage adherent to ART Black:White MSM | 0.82-1 | US cohort data and US HIV surveillance[33,35,44-47] |
| $\phi_{5,1}\phi_{6,1}\phi_{7,1}$ | Dropout from ART, White MSM, not fully suppressed/1^st^ year on ART/2^nd^ year on ART, per year | 0.06-0.13 | Rate of dropout from ART, US [35-39] |
| $\phi_{z ratio}$ | Ratio of dropout from ART 3^rd^+ years: dropout 1^st^, 2^nd^ years ($\phi_{8}: \phi_{5}$) | 0.5-1.0 | Rate of dropout from US ART cohorts [48] |
| $\phi_{w ratio}$ | Ratio of ART dropout for Black:White MSM | 0.7-1.6 | US ART cohort data[38,48] |
| $\zeta$ | Rate of re-enrolment into pre-ART HIV care for those dropping out of ART, per year | 0.05-1 | From rate of dropout and re-joining US ART cohorts [48] |
| $s_{n,0}$ | Percentage of Black MSM circumcised | 75-87 | NHBS 2014 |
| $s_{n,1}$ | Percentage of White MSM circumcised | 87-96 | NHBS 2014 |
| $\delta_{1}$ | Relative rate of being offered PrEP: testing for HIV, for those with a PrEP indication, 2020-2021^b^ | 2-6 | Range explored in base case scenario; rate increases linearly from 0 in 2012. Rate varied from 2022 in long-acting PrEP scenarios. |
| $\delta_{0}$ | Relative rate of being offered PrEP: testing for HIV, for those without a PrEP indication, 2020-2021 | 0 | In base case scenario, assume no PrEP initiations by those without a PrEP indication. Non-zero values used from 2022 in long-acting PrEP scenarios with proportionate PrEP distribution, or with eligible PrEP distribution where additional PrEP users were required to meet the PrEP coverage target. |
| $o_{0,0}$ | PrEP acceptance (% accepting PrEP when offered), 18-24 year-old Black MSM | 40.8-64.2 (mode 52.6)^d^ | Stratified analysis of US PrEP Demo project data |
| $o_{0,1}$ | PrEP acceptance (% accepting PrEP when offered), 18-24 year-old White MSM | 64.6-74.0 (mode 69.5)^d^ | Stratified analysis of US PrEP Demo project data |
| $o_{1,0}$ | PrEP acceptance (% accepting PrEP when offered), >24 year-old Black MSM | 40.8-64.2 (mode 52.6)^d^ | Stratified analysis of US PrEP Demo project data |
| $o_{1,1}$ | PrEP acceptance (% accepting PrEP when offered), >24 year-old White MSM | 64.6-74.0 (mode 69.5) ^d^ | Stratified analysis of US PrEP Demo project data |
| $s_{ph0,0}$ | Adherence to daily oral PrEP with TDF/FTC (% taking ≥4 doses/week), 18-24 year old Black MSM | 47.2-82.7 (mode 67.3) ^d^ | Stratified analysis of US PrEP Demo project data |
| $s_{ph0,1}$ | Adherence to daily oral PrEP with TDF/FTC (% taking ≥4 doses/week), 18-24 year old White MSM | 85.8-97.5 (mode 93.2) ^d^ | Stratified analysis of US PrEP Demo project data |
| $s_{ph1,0}$ | Adherence to daily oral PrEP with TDF/FTC (% taking ≥4 doses/week), >24 year-old Black MSM | 43.7-64.2 (mode 54.1) ^d^ | Stratified analysis of US PrEP Demo project data |
| $s_{ph1,1}$ | Adherence to daily oral PrEP with TDF/FTC (% taking ≥4 doses/week), >24 year-old White MSM | 87.7-92.7 (mode 90.4) ^d^ | Stratified analysis of US PrEP Demo project data |
| $s_{pm0,0}$, $s_{pm0,1}$,$s_{pm1,0}$,$s_{pm1,1}$ | % of those taking <4 doses/week who take 2-4 doses/week of daily oral TDF/FTC | 45 (fixed) | Percentage estimated from full followup (of up to 81 weeks) of randomly selected adherence subset of MSM in the HPTN 083 trial[49] |
| $\iota_{1,0,0}$ | PrEP dropout rate per person per year for oral TDF/FTC PrEP, 18-24 year old Black MSM | 0.280-0.849  (mode 0.511) ^d^ | Rate calculated from proportion not retained at the end of the study, stratified analysis of US PrEP Demo project data |
| $\iota_{1,0,1}$ | PrEP dropout rate per person per year for oral TDF/FTC PrEP, 18-24 year old White MSM | 0.033-0.357  (mode 0.190) ^d^ | Rate calculated from proportion not retained at the end of the study, stratified analysis of US PrEP Demo project data |
| $\iota_{1,1,0}$ | PrEP dropout rate per person per year for oral TDF/FTC PrEP, >24 year-old Black MSM | 0.280-0.849  (mode 0.511) ^d^ | Rate calculated from proportion not retained at the end of the study, stratified analysis of US PrEP Demo project data |
| $\iota_{1,1,1}$ | PrEP dropout rate per person per year for oral TDF/FTC PrEP, >24 year-old White MSM | 0.151-0.270  (mode 0.216) ^d^ | Rate calculated from proportion not retained at the end of the study, stratified analysis of US PrEP Demo project data |
| ${\iota_{2,0,0,} \iota}_{2,0,1,}\iota_{2,1,0,}$  $\iota_{2,1,1}$ | PrEP dropout rate per person per year for long-acting injectable CAB, all age and race groups | 0.184 (fixed) | Fixed at value for US participants in the HPTN 083 trial for this model comparison |
| ***Intervention efficacy*** | | | |
| $e_{c}$ | Reduction in HIV acquisition risk due to correct condom use (%) | 58-79 | Estimate for US MSM[50] |
| $e_{n}$ | Reduction in HIV acquisition risk due to male circumcision (%) | 12-23 | Assuming same efficacy as for heterosexual men from RCTs[51], only protective in insertive acts, half of all sex acts are insertive, receptive sex acts carry a 2.3x higher risk of transmission than insertive[24] |
| $d_{r}$ | Relative level of infectiousness of those on ART and partially suppressed, scaled between the level for those fully suppressed ${(d}_{r}=0$) and those unsuppressed ($d_{r}=1)$ | 0.5 (fixed) | assumption |
| $d_{6}$ | Relative level of infectiousness of those on ART and fully suppressed vs chronic infection untreated (CD4>200) | 0 (fixed) | Equivalent to 100% reduction in HIV transmission. Estimates from discordant MSM partnerships where HIV-positive partner on ART and virally suppressed[52] |
| $e_{ph}$ | Reduction in HIV acquisition risk when adherent to daily oral PrEP with TDF/FTC (taking ≥4 tablets/week) | 90-100 (mode 96) ^d^ | Efficacy estimated by Anderson et al from iPrEx and STRAND trial data analysis for 4 doses/week[53] |
| $e_{pm}$ | Reduction in HIV acquisition risk when partially adherent to daily oral PrEP with TDF/FTC (taking 2-4 tablets/week) | 56-96 (mode 76) ^d^ | Efficacy estimated by Anderson et al from iPrEx and STRAND trial data analysis for 2 doses/week[53] |
| $e_{pi}$ | Reduction in HIV acquisition risk when using long-acting injectable CAB (including those not adhering to injection schedule) | 82-96 (mode 91)^d^ | Effectiveness (efficacy × adherence) estimated from HPTN 083 trial data |

^a^Limits of uniform/triangular prior distribution

^b^Final values for time-varying parameters. Earlier values or earlier gradient of parameter function given elsewhere in table S1.

^c^Guideline changes coded in: pre-1996, no initiation of ART [54] From 1996-1998 ART initiation at any CD4 count; from 1998-Feb 2001, initiation from care with CD4<500 (1998 guidelines); from Feb 2001-Dec 2009 initiation with CD4 <350 (2001 guidelines); from Dec 2009-March 2012 initiation from care with CD4<500 (2009 guidelines); from March 2012 onwards initiation from care with any CD4 count (2012 guidelines). These apply to all age and race groups.

^d^Triangular distribution used (mode given in brackets)

***References***

1. Purcell DW, Johnson CH, Lansky A, Prejean J, Stein R, Denning P, et al. Estimating the population size of men who have sex with men in the United States to obtain HIV and syphilis rates. Open AIDS J. 2012;6:98-107.

2. Grey JA, Bernstein KT, Sullivan PS, Purcell DW, Chesson HW, Gift TL, et al. Estimating the population sizes of men who have sex with men in US states and counties using data from the American community survey. JMIR Public Health Surveill. 2016;2(1):e14.

3. CDC. WONDER online database [Available from: <https://wonder.cdc.gov/>.

4. Sullivan PS, Salazar L, Buchbinder S, Sanchez TH. Estimating the proportion of HIV transmissions from main sex partners among men who have sex with men in five US cities. AIDS. 2009;23(9):1153-62.

5. Delaney KP, Rosenberg ES, Kramer MR, Waller LA, Sullivan PS. Optimizing human immunodeficiency virus testing interventions for men who have sex with men in the United States: a modeling study. Open Forum Infect Dis. 2015;2(4):ofv153.

6. Mitchell JW, Petroll AE. Patterns of HIV and sexually transmitted infection testing among men who have sex with men couples in the United States. Sex Transm Dis. 2012;39(11):871-6.

7. Silhol R, Boily M-C, Dimitrov D, German D, Flynn C, Farley JE, et al. Understanding the HIV epidemic among MSM in Baltimore: A modeling study estimating the impact of past HIV interventions and who acquired and contributed to infections. J Acquir Immune Defic Syndr. 2020;84(3):253-62.

8. Kalichman SC, Eaton L, Cain D, Cherry C, Fuhrel A, Kaufman M, et al. Changes in HIV treatment beliefs and sexual risk behaviors among gay and bisexual men, 1997-2005. Health Psychol. 2007;26(5):650-6.

9. Hollingsworth TD, Anderson RM, Fraser C. HIV-1 transmission, by stage of infection. J Infect Dis. 2008;198(5):687-93.

10. Boily MC, Baggaley RF, Wang L, Masse B, White RG, Hayes RJ, et al. Heterosexual risk of HIV-1 infection per sexual act: systematic review and meta-analysis of observational studies. Lancet Infect Dis. 2009;9(2):118-29.

11. Dunn D, Woodburn P, Duong T, Peto J, Phillips A, Gibb D, et al. Current CD4 cell count and the short-term risk of AIDS and death before the availability of effective antiretroviral therapy in HIV-infected children and adults. J Infect Dis. 2008;197(3):398-404.

12. Cori A, Pickles M, van Sighem A, Gras L, Bezemer D, Reiss P, et al. CD4+ cell dynamics in untreated HIV-1 infection: overall rates, and effects of age, viral load, sex and calendar time. AIDS. 2015;29(18):2435-46.

13. Egger M, May M, Chene G, Phillips AN, Ledergerber B, Dabis F, et al. Prognosis of HIV-1-infected patients starting highly active antiretroviral therapy: a collaborative analysis of prospective studies. Lancet. 2002;360(9327):119-29.

14. UK Office for National Statistics. Deaths: age sex. England and Wales [table 6.1]. Popul Trends. 2006;126:49.

15. Antiretroviral Therapy Cohort Collaboration. Importance of baseline prognostic factors with increasing time since initiation of highly active antiretroviral therapy: collaborative analysis of cohorts of HIV-1-infected patients. J Acquir Immune Defic Syndr. 2007;46(5):607-15.

16. Michigan Department of Community Health. Adult and Adolescent Spectrum of Disease Project in Michigan Summary Report 1990-2003.

17. Mellors JW, Munoz A, Giorgi JV, Margolick JB, Tassoni CJ, Gupta P, et al. Plasma viral load and CD4+ lymphocytes as prognostic markers of HIV-1 infection. Ann Intern Med. 1997;126(12):946-54.

18. Herbeck JT, Gottlieb GS, Li X, Hu Z, Detels R, Phair J, et al. Lack of evidence for changing virulence of HIV-1 in North America. PloS one. 2008;3(2):e1525.

19. Drake AL, Kinuthia J, Matemo D, McClelland RS, Richardson BA, Overbaugh J, et al. Virologic and immunologic response following antiretroviral therapy initiation among pregnant and postpartum women with acute HIV-1 infection [abstract #MOPDB0101]. International AIDS conference; Melbourne, Australia2014.

20. Mitchell KM, Hoots B, Dimitrov D, German D, Flynn C, Farley JE, et al. Improvements in the HIV care continuum needed to meaningfully reduce HIV incidence among men who have sex with men in Baltimore, US: a modelling study for HPTN 078. J Int AIDS Soc. 2019;22(3):e25246.

21. Donnell D, Baeten JM, Kiarie J, Thomas KK, Stevens W, Cohen CR, et al. Heterosexual HIV-1 transmission after initiation of antiretroviral therapy: a prospective cohort analysis. Lancet. 2010;375(9731):2092-8.

22. Hallett TB, Baeten JM, Heffron R, Barnabas R, de Bruyn G, Cremin Í, et al. Optimal uses of antiretrovirals for prevention in HIV-1 serodiscordant heterosexual couples in South Africa: a modelling study. PLoS Med. 2011;8(11):e1001123.

23. Baggaley RF, White RG, Boily MC. HIV transmission risk through anal intercourse: systematic review, meta-analysis and implications for HIV prevention. Int J Epidemiol. 2010;39(4):1048-63.

24. Jin F, Jansson J, Law M, Prestage GP, Zablotska I, Imrie JC, et al. Per-contact probability of HIV transmission in homosexual men in Sydney in the era of HAART. AIDS. 2010;24(6):907-13.

25. Blaser N, Wettstein C, Estill J, Vizcaya LS, Wandeler G, Egger M, et al. Impact of viral load and the duration of primary infection on HIV transmission: systematic review and meta-analysis. AIDS. 2014;28(7):1021-9.

26. Anderson JE, Carey JW, Taveras S. HIV testing among the general US population and persons at increased risk: information from national surveys, 1987-1996. Am J Public Health. 2000;90(7):1089-95.

27. Centers for Disease Control and Prevention: US Public Health Service. Preexposure prophylaxis for the prevention of HIV infection in the United States—2017 Update: a clinical practice guideline. . 2018.

28. Georgia Department of Public Health. HIV/AIDS Epidemiology Program HIV Care Continuum Surveillance Report, Georgia, 2012. 2014.

29. Georgia Department of Public Health. HIV Care Continuum Report, Georgia, 2014. 2016.

30. Centers for Disease Control and Prevention. Reported CD4+ T-lymphocyte results for adults and adolescents with HIV/AIDS - 33 states, 2005. HIV/AIDS Surveillance Report. 2005;11(2).

31. Centers for Disease Control and Prevention. Reported CD4+ T-lymphocyte and viral load results for adults and adolesecents with HIV infection - 37 states, 2005-2007. HIV Surveillance Supplemental Report. 2010;16(1).

32. Georgia Department of Public Health. HIV/AIDS epidemiology program HIV care continuum surveillance report, Georgia, 2011. 2013.

33. Singh S, Bradley H, Hu X, Skarbinski J, Hall HI, Lansky A. Men living with diagnosed HIV who have sex with men: progress along the continuum of HIV care--United States, 2010. MMWR Morb Mortal Wkly Rep. 2014;63(38):829-33.

34. Hoots BE, Finlayson TJ, Wejnert C, Paz-Bailey G. Early linkage to HIV care and antiretroviral treatment among men who have sex with men - 20 cities, United States, 2008 and 2011. PLoS One. 2015;10(7):e0132962.

35. Moore RD, Keruly JC, Bartlett JG. Improvement in the health of HIV-infected persons in care: reducing disparities. Clin Infect Dis. 2012;55(9):1242-51.

36. Rebeiro P, Althoff KN, Buchacz K, Gill J, Horberg M, Krentz H, et al. Retention among North American HIV-infected persons in clinical care, 2000-2008. J Acquir Immune Defic Syndr. 2013;62(3):356-62.

37. Tedaldi EM, Richardson JT, Debes R, Young B, Chmiel JS, Durham MD, et al. Retention in care within 1 year of initial HIV care visit in a multisite US cohort: who's in and who's out? J Int Assoc Provid AIDS Care. 2014;13(3):232-41.

38. Li X, Margolick JB, Conover CS, Badri S, Riddler SA, Witt MD, et al. Interruption and discontinuation of highly active antiretroviral therapy in the multicenter AIDS cohort study. J Acquir Immune Defic Syndr. 2005;38(3):320-8.

39. Howe CJ, Cole SR, Napravnik S, Eron JJ. Enrollment, retention, and visit attendance in the University of North Carolina Center for AIDS Research HIV clinical cohort, 2001-2007. AIDS Res Hum Retroviruses. 2010;26(8):875-81.

40. Dasgupta S, Oster AM, Li J, Hall HI. Disparities in consistent retention in HIV care - 11 states and the district of Columbia, 2011-2013. MMWR Morb Mortal Wkly Rep. 2016;65(4):77-82.

41. Cohen MS, Chen YQ, McCauley M, Gamble T, Hosseinipour MC, Kumarasamy N, et al. Antiretroviral therapy for the prevention of HIV-1 transmission. N Engl J Med. 2016;375(9):830-9.

42. Guiguet M, Porter K, Phillips A, Costagliola D, Babiker A. Clinical progression rates by CD4 cell category before and after the initiation of combination antiretroviral therapy (cART). Open AIDS J. 2008;2:3-9.

43. Mocroft A, Furrer HJ, Miro JM, Reiss P, Mussini C, Kirk O, et al. The incidence of AIDS-defining illnesses at a current CD4 count >/= 200 cells/muL in the post-combination antiretroviral therapy era. Clin Infect Dis. 2013;57(7):1038-47.

44. Althoff KN, Rebeiro P, Brooks JT, Buchacz K, Gebo K, Martin J, et al. Disparities in the quality of HIV care when using US Department of Health and Human Services indicators. Clin Infect Dis. 2014;58(8):1185-9.

45. Novak RM, Hart RL, Chmiel JS, Brooks JT, Buchacz K. Disparities in initiation of combination antiretroviral treatment and in virologic suppression among patients in the HIV Outpatient Study (HOPS), 2000-2013. J Acquir Immune Defic Syndr. 2015.

46. Weintrob AC, Grandits GA, Agan BK, Ganesan A, Landrum ML, Crum-Cianflone NF, et al. Virologic response differences between African Americans and European Americans initiating highly active antiretroviral therapy with equal access to care. J Acquir Immune Defic Syndr. 2009;52(5):574-80.

47. Robertson M, Laraque F, Mavronicolas H, Braunstein S, Torian L. Linkage and retention in care and the time to HIV viral suppression and viral rebound - New York City. AIDS Care. 2015;27(2):260-7.

48. Krishnan S, Wu K, Smurzynski M, Bosch RJ, Benson CA, Collier AC, et al. Incidence rate of and factors associated with loss to follow-up in a longitudinal cohort of antiretroviral-treated HIV-infected persons: an AIDS Clinical Trials Group (ACTG) Longitudinal Linked Randomized Trials (ALLRT) analysis. HIV Clin Trials. 2011;12(4):190-200.

49. Landovitz RJ, Donnell D, Clement ME, Hanscom B, Cottle L, Coelho L, et al. Cabotegravir for HIV Prevention in Cisgender Men and Transgender Women. N Engl J Med. 2021;385(7):595-608.

50. Smith DK, Herbst JH, Zhang X, Rose CE. Condom effectiveness for HIV prevention by consistency of use among men who have sex with men in the United States. J Acquir Immune Defic Syndr. 2015;68(3):337-44.

51. Mills E, Cooper C, Anema A, Guyatt G. Male circumcision for the prevention of heterosexually acquired HIV infection: a meta-analysis of randomized trials involving 11,050 men. HIV Med. 2008;9(6):332-5.

52. Rodger AJ, Cambiano V, Bruun T, Vernazza P, Collins S, van Lunzen J, et al. Sexual activity without condoms and risk of HIV transmission in serodifferent couples when the HIV-positive partner is using suppressive antiretroviral therapy. JAMA. 2016;316(2):171-81.

53. Anderson PL, Glidden DV, Liu A, Buchbinder S, Lama JR, Guanira JV, et al. Emtricitabine-tenofovir concentrations and pre-exposure prophylaxis efficacy in men who have sex with men. Sci Transl Med. 2012;4(151):151ra25.

54. Palella FJ, Jr., Delaney KM, Moorman AC, Loveless MO, Fuhrer J, Satten GA, et al. Declining morbidity and mortality among patients with advanced human immunodeficiency virus infection. HIV Outpatient Study Investigators. N Engl J Med. 1998;338(13):853-60.

**B. The Montreal Model**

**Table S3:** Model parameters and their prior distributions, if applicable. References to additional tables can be found in the Milwid et al. 2022 Supplementary Materials. *From:* *Milwid RM, Xia Y, Doyle CM, Cox J, Lambert G, Thomas R, Mishra S, Grace D, Lachowsky NJ, Hart TA, Boily MC, Maheu-Giroux M. Past dynamics of HIV transmission among men who have sex with men in Montréal, Canada: a mathematical modeling study. BMC Infect Dis. 2022 Mar 7;22(1):233. doi: 10.1186/s12879-022-07207-7. PMID: 35255860; PMCID: PMC8902714. (Supplementary Materials.)*

| Parameter | Value | Prior distribution for calibrated parameters | Reference |
| --- | --- | --- | --- |
| Demographic parameters | | | |
| Initial model population size | 10,000 |  |  |
| Annual population growth rate | See Table 2 | fixed | (32, 33, 36) |
| Initial age distribution | See Table 3 | fixed | (35) |
| HIV prevalence in 1975 |  | ~U(0.02- 0.50%) | Assumed |
| Sexual behaviours | | | |
| Proportion of men whose sexual preference is “versatile” | 0.64 | fixed | *Engage* |
| Proportion of men whose sexual preference is “receptive” | 0.17 | fixed |  |
| Proportion of men whose sexual preference is “insertive” | 0.20 | fixed |  |
| Proportion of regular partnerships (>1 month) $\boldsymbol{(}\boldsymbol{p}_{\boldsymbol{regular}}\boldsymbol{)}$ |  | ~U(0.0 - 0.4) | Informed from *Argus I, Argus II*, and *Engage* |
| Number of anal sex partners per 6-month-period for the referent age group (15-20 years) |  | $\sim NB\left( \mu,s \right)$  $\mu$= [3.7 – 4.18]  $s= \left[ 0.56 - 1.29 \right]$ | *Engage Argus II Argus I* |
| Partner change rate scaling factor for men aged 20-24 years ($\boldsymbol{R}\boldsymbol{R}_{\boldsymbol{22-24}}$) | 1.22 | fixed |  |
| Partner change rate scaling factor for men aged 25-29 years ($\boldsymbol{R}\boldsymbol{R}_{\boldsymbol{25-29}}$) | 1.41 | fixed |  |
| Partner change rate scaling factor for men aged 30-34 years ($\boldsymbol{R}\boldsymbol{R}_{\boldsymbol{30-34}}$) | 1.54 | fixed |  |
| Partner change rate scaling factor for men aged 35-39 years ($\boldsymbol{R}\boldsymbol{R}_{\boldsymbol{35-39}}$) | 1.60 | fixed |  |
| Partner change rate scaling factor for men aged 40-44 years ($\boldsymbol{R}\boldsymbol{R}_{\boldsymbol{40-44}}$) | 1.56 | fixed |  |
| Partner change rate scaling factor for men aged 45-49 years ($\boldsymbol{R}\boldsymbol{R}_{\boldsymbol{45-49}}$) | 1.44 | fixed |  |
| Partner change rate scaling factor for men aged 50-54 years ($\boldsymbol{R}\boldsymbol{R}_{\boldsymbol{50-54}}$) | 1.26 | fixed |  |
| Partner change rate scaling factor for men aged 55-59 years ($\boldsymbol{R}\boldsymbol{R}_{\boldsymbol{55-59}}$) | 1.04 | fixed |  |
| Partner change rate scaling factor for men aged 60-64 years ($\boldsymbol{R}\boldsymbol{R}_{\boldsymbol{60-64}}$) | 0.81 | fixed |  |
| Partner change rate scaling factor for men aged 65-69 years ($\boldsymbol{R}\boldsymbol{R}_{\boldsymbol{65-69}}$) | 0.60 | fixed |  |
| Partner change rate scaling factor for men aged 70+ years ($\boldsymbol{R}\boldsymbol{R}_{\boldsymbol{70-}}\boldsymbol{)}$ | 0.42 | fixed |  |
| Duration of a regular partnership  ($\boldsymbol{duratio}{\boldsymbol{n}_{\boldsymbol{regular}}}\boldsymbol{)}$ |  | $\sim NB\left( \mu,s \right)$  $\mu$= [1.00- 55.81]  s= [0.83 - 0.94] |  |
| Number of anal sex acts per casual partnership | 1-3 | Not calibrated |  |
| Number of anal sex acts over a six month period per regular partnership (Poisson distributed) |  | $\lambda=$ [20.49 – 22.35] | *Engage* |
| HIV-disease progression parameters | | | |
| Per-act probability of HIV acquisition for insertive anal intercourse  $\boldsymbol{(}\boldsymbol{p}_{\boldsymbol{insertive}}\boldsymbol{)}$ |  | ~U(0.04-0.28%) | (86) |
| Relative risk of HIV acquisition for receptive anal intercourse $\boldsymbol{(R}\boldsymbol{R}_{\boldsymbol{receptive}}\boldsymbol{)}$ | 3.846 | fixed | (86) |
| Relative risk of HIV transmission during the acute acquisition stage ($\boldsymbol{R}\boldsymbol{R}_{\boldsymbol{CD}\boldsymbol{4}_{\boldsymbol{1}}}\boldsymbol{)}$ | 9.17 | fixed | (87) |
| Relative risk of HIV transmission when CD4^+^cell counts < 200 $\boldsymbol{cells \mu}\boldsymbol{L}^{\boldsymbol{-1}}$ $\boldsymbol{(R}\boldsymbol{R}_{\boldsymbol{CD}\boldsymbol{4}_{\boldsymbol{2}}}\boldsymbol{)}$ | 7.27 | fixed |  |
| Fraction of individuals who transition from primary stage of acquisition to a CD4 cell count > 500 $\boldsymbol{cells \mu}\boldsymbol{L}^{\boldsymbol{-1}}$ ($\boldsymbol{p}_{\boldsymbol{0}}$) | 76% | fixed | (54) |
| Fraction of individuals going from primary stage to a CD4 cell count between 350 and 500$\boldsymbol{cells \mu}\boldsymbol{L}^{\boldsymbol{-1}}$($\boldsymbol{p}_{\boldsymbol{1}}$) | 19% | fixed |  |
| Fraction of individuals going from primary stage to a CD4 cell count between 200 and 350$\boldsymbol{cells \mu}\boldsymbol{L}^{\boldsymbol{-1}}$($\boldsymbol{p}_{\boldsymbol{2}}$) | 5% | fixed |  |
| Transition rate from the primary stage of acquisition to a CD4 cell count > 500 $\boldsymbol{cells \mu}\boldsymbol{L}^{\boldsymbol{-1}}$  ($\boldsymbol{\gamma}_{\boldsymbol{1}}\boldsymbol{)}$ | $\frac{1}{0.25}$ year^-1^ | fixed |  |
| Transition rate from a CD4 cell count > 500 $\boldsymbol{cells \mu}\boldsymbol{L}^{\boldsymbol{-1}}$ to a CD4 cell count between 350 and 500$\boldsymbol{cells \mu}\boldsymbol{L}^{\boldsymbol{-1}}$  ($\boldsymbol{\gamma}_{\boldsymbol{2}}\boldsymbol{)}$ | $\frac{1}{3.32}$year^-1^ | fixed |  |
| Transition rate from a CD4 cell count between 350 and 500 $\boldsymbol{cells \mu}\boldsymbol{L}^{\boldsymbol{-1}}$ to a CD4 cell count between 200 and 350$\boldsymbol{cells \mu}\boldsymbol{L}^{\boldsymbol{-1}}$ ($\boldsymbol{\gamma}_{\boldsymbol{3}}\boldsymbol{)}$ | $\frac{1}{2.70}$year^-1^ | fixed |  |
| Transition rate from a CD4 cell count between 200 $\boldsymbol{cells \mu}\boldsymbol{L}^{\boldsymbol{-1}}$ and 350 $\boldsymbol{cells \mu}\boldsymbol{L}^{\boldsymbol{-1}}$ to a CD4 cell count <200$\boldsymbol{cells \mu}\boldsymbol{L}^{\boldsymbol{-1}}$  ($\boldsymbol{\gamma}_{\boldsymbol{4}}\boldsymbol{)}$ | $\frac{1}{5.50}$year^-1^ | fixed |  |
| HIV and AIDS related death rate ( $\boldsymbol{p}_{\boldsymbol{death\_age\_CD}\boldsymbol{4}}\boldsymbol{)}$ | See Tables 6 and 7 | fixed | (56) |
| Interventions | | | |
| Testing | | | |
| Proportion of men who are symptomatic when CD4 cell counts are <200$\boldsymbol{cells \mu}\boldsymbol{L}^{\boldsymbol{-1}}$  ($\boldsymbol{\rho}_{\boldsymbol{t,2}}\boldsymbol{)}$ | 93% | fixed | (88) |
| Proportion of men on PrEP who get tested every three months $\boldsymbol{(\rho}_{\boldsymbol{PrEP}}\boldsymbol{)}$ | 100% | fixed | Assumed |
| Proportion of men on PEP who get tested at 1 and 3 months after PEP initiation | 68% | fixed | (70) |
| Proportion of men ever tested for HIV | See Table 9 |  |  |
| Annual proportion of men tested for HIV | See Table 10 |  |  |
| HIV testing rate for the low partnering group  $\boldsymbol{(}\boldsymbol{\rho}_{\boldsymbol{tes}\boldsymbol{t}_{\boldsymbol{t}}\boldsymbol{l,}}\boldsymbol{)}$ | See Table 11 |  | *Engage Argus II Argus I* |
| Minimum time (months) between HIV tests |  | $\sim ZTPoisson\left( \lambda\right)$  $\lambda=\frac{1}{\rho_{test_{t}}}$ | Interpolation *Argus II* *Argus I Engage* |
| HIV test sensitivity and specificity | 100% | fixed | (67) |
| Testing interval for individuals on PrEP | 3 months | fixed | Assumed |
| Rate ratio for HIV testing rate ($\boldsymbol{\rho}_{\boldsymbol{tes}\boldsymbol{t}_{\boldsymbol{t}}}\boldsymbol{)}$ among the combined medium and high partnering individuals (>5partners per annum)  ($\boldsymbol{R}\boldsymbol{R}_{\boldsymbol{test}}$) | 1.46 | fixed | Calculated from the combined *Argus I*, *Argus II*, and *Engage* data |
| Period between HIV acquisition and the ability to detect HIV | See Table 8 |  | (72) |
| Treatment | | | |
| ART efficacy (for partial viral load suppression)  ($\boldsymbol{\epsilon}_{\boldsymbol{ART\_sART}}$) | 80% | fixed | (51) |
| ART efficacy (for full viral load suppression)  ($\boldsymbol{\epsilon}_{\boldsymbol{ART\_lART}}$) |  | ~U(90-100%) | (21, 49) |
| Time delay (months) between HIV diagnosis and ART uptake (for diagnosis prior to 1996)  ($\boldsymbol{\theta}_{\boldsymbol{1996}}\boldsymbol{)}$ |  | $\sim NB\left( \mu,s \right)$  s = [0.10, 3.11]  $\mu$= [3.65, 6.80] | *Argus I*  *Engage* |
| Time delay (months)between HIV diagnosis and ART uptake (for diagnosis between 1996-2004)  ($\boldsymbol{\theta}_{\boldsymbol{1996-2004}}\boldsymbol{)}$ |  | $\sim NB\left( \mu,s \right)$  s = [0.14, 0.28]  μ= [12.30, 41.19] |  |
| Time delay (months)between HIV diagnosis and ART uptake (for diagnosis between 2004-2012)  ($\boldsymbol{\theta}_{\boldsymbol{2004-2012}}\boldsymbol{)}$ |  | $\sim NB\left( \mu,s \right)$  s = [0.06, 0.24]  $\mu$= [1.79, 10.11] |  |
| Time delay (months)between HIV diagnosis and ART uptake (for diagnosis after 2012) ($\boldsymbol{\theta}_{\boldsymbol{2012}}\boldsymbol{)}$ | Instantaneous | fixed |  |
| ART discontinuation rate  ($\boldsymbol{\psi}$) |  | ~U$\left( \frac{1}{113.91}, \frac{1}{89.40} \right)$ month^-1^ | Argus II |
| ART re-initiation rate (2005) $\boldsymbol{(}\boldsymbol{\delta}_{\boldsymbol{2005}}\boldsymbol{)}$ |  | ~U($\frac{1}{32.71},\frac{1}{5.4})$ month^-1^ | *Argus I* |
| ART re-initiation rate (2017) $\boldsymbol{(}\boldsymbol{\delta}_{\boldsymbol{2017}}\boldsymbol{)}$ |  | ~U($\frac{1}{3.28},\frac{1}{1.1}$) month^-1^ | *Engage* |
| Duration of short-term ART user categorization ($\boldsymbol{\phi)}$ | 3-6 months | Not calibrated | (74) |
| Condom use | | | |
| Condom efficacy | 91% | fixed | (76) |
| Per partner probability of condom use  (${\boldsymbol{P}_{\boldsymbol{condoms}}}_{\boldsymbol{t, partnership\_type}}\boldsymbol{)}$ | See Table 12 | fitted | *Engage Argus II Argus I Omega*  (77, 78) |
| Per act probability of condom use (${\boldsymbol{P}_{\boldsymbol{act\_condoms}}}_{\boldsymbol{t, partnership\_type}}\boldsymbol{)}$ | See 13 | fixed | *Engage Argus II Argus I* |
| Pre-exposure prophylaxis (PrEP) | | | |
| PrEP efficacy ($\boldsymbol{\epsilon}_{\boldsymbol{PrEP}}$) | 86% | fixed | (81) |
| Proportion of all eligible men who initiate PrEP annually (2013 - 2014)  ($\boldsymbol{\vartheta}_{\boldsymbol{PrEP}_{\boldsymbol{2013}}}\boldsymbol{)}$ |  | ~U(0, 0.01) | *Engage* |
| Proportion of all eligible men who initiate PrEP annually (2015)  ($\boldsymbol{\vartheta}_{\boldsymbol{PrEP}_{\boldsymbol{2015}}}\boldsymbol{)}$ |  | ~U(0.01, 0.24) | *Engage* |
| Proportion of all eligible men who initiate PrEP annually (2016)  ($\boldsymbol{\vartheta}_{\boldsymbol{PrEP}_{\boldsymbol{2016}}}\boldsymbol{)}$ |  | ~U(0.01, 0.17) | *Engage* |
| Proportion of all eligible men who initiate PrEP annually (2017)  ($\boldsymbol{\vartheta}_{\boldsymbol{PrEP}_{\boldsymbol{2017}}}\boldsymbol{)}$ |  | ~U(0.01, 0.26) | *Engage* |
| Proportion of all eligible men who initiate PrEP annually (2018)  ($\boldsymbol{\vartheta}_{\boldsymbol{PrEP}_{\boldsymbol{2018}}}\boldsymbol{)}$ |  | ~U(0.06, 0.32) | *Engage* |
| Proportion of all eligible men who initiate PrEP annually (2019)  ($\boldsymbol{\vartheta}_{\boldsymbol{PrEP}_{\boldsymbol{2019}}}\boldsymbol{)}$ |  | ~U(0.06, 0.32) | *Engage* |
| PrEP discontinuation rate |  | ~U($\frac{1}{8.44},\frac{1}{13.00}$) month^-1^ | *l’Actuel* |
| Post-exposure prophylaxis (PEP) | | | |
| PEP efficacy $\boldsymbol{(}\boldsymbol{\epsilon}_{\boldsymbol{PEP}}\boldsymbol{)}$ | 80% | fixed | (89) |
| Proportion of all eligible men who attempted PEP use in 2000   ($\boldsymbol{\rho}_{\boldsymbol{PE}\boldsymbol{P}_{\boldsymbol{2001}}}\boldsymbol{)}$ | 0% | fixed | assumed |
| Proportion of all eligible men who attempted PEP use in 2017 onwards  ($\boldsymbol{\rho}_{\boldsymbol{PE}\boldsymbol{P}_{\boldsymbol{2017}}}\boldsymbol{)}$ |  | ~U(0.03, 0.11) | *Engage* |

***References***

21. Rodger AJ, Cambiano V, Bruun T, Vernazza P, Collins S, van Lunzen J, et al. Sexual activity without condoms and risk of HIV transmission in serodifferent couples when the HIV-positive partner is using suppressive antiretroviral therapy. JAMA. 2016;316(2):171-81.

32. Statistics Canada. Census Canada 1986. 1987.

33. Statistics Canada. 1991 Census of population. 1991.

35. Statistics Canada. 1976 Census of Canada. Volume 2, population, demographic characteristics. 1976.

36. Institut de la statistique du Québec. Estimations de la population des MRC selon le groupe d'âge et le sexe, âge médian et âge moyen, Québec, 1er juillet 1996 à 2018. 2020.

49. Cohen MS, Chen YQ, McCauley M, Gamble T, Hosseinipour MC, Kumarasamy N, et al. Antiretroviral therapy for the prevention of HIV-1 transmission. N Engl J Med. 2016;375(9):830-9.

51. Wilson DP, Law MG, Grulich AE, Cooper DA, Kaldor JM. Relation between HIV viral load and infectiousness: a model-based analysis. The Lancet. 2008;372(9635):314-20.

54. Cori A, Pickles M, van Sighem A, Gras L, Bezemer D, Reiss P, et al. CD4+ cell dynamics in untreated HIV-1 infection: overall rates, and effects of age, viral load, sex and calendar time. AIDS. 2015;29(18):2435-46.

56. Avenir Health. Spectrum system of policy models 2019 [Available from: https://www.avenirhealth.org/software-spectrum.php.

67. Kimmig P. Sensitivity and specificity of test results in HIV. Das Offentliche Gesundheitswesen. 1990;52(8-9):419 - 24.

70. Xia Y, Greenwald ZR, Milwid RM, Claire T, Boissonnault M, Gaul N, et al. Pre-exposure prophylaxis uptake among men who have sex with men who used non-occupational post-exposure prophylaxis: a longitudinal analysis of attendees at a large sexual health clinic in Montreal (Canada). J Acquir Immune Defic Syndr. 2020.

72. Alexander TS. Human immunodeficiency virus diagnostic testing: 30 years of evolution. Clin Vaccine Immunol. 2016;23(4):249-53.

74. Kasaie P, Pennington J, Shah MS, Berry SA, German D, Flynn CP, et al. The impact of preexposure prophylaxis among men who have sex with men: An individual-based model. J Acquir Immune Defic Syndr. 2017;75(2):175-83.

76. Johnson WD, O'Leary A, Flores SA. Per-partner condom effectiveness against HIV for men who have sex with men. AIDS. 2018;32(11):1499-505.

77. Lévy JJ, Dupras A, Doras M, Perreault M, Frigault L-R. Styles de relations et conduites sexuelles d’hommes francophones montréalais qui ont des rapports sexuels avec d’autres hommes. Service social. 1996;45(1).

78. Dufour A, Alary M, Otis J, Remis RS, Masse Bit, Turmel B, et al. Risk behaviours and HIV infection among men having sexual relations with men: baseline characteristics of participants in the Omega Cohort Study, Montreal, Quebec, Canada. Canadian Journal of Public Health. 2000;91(5):345--9.

81. Molina JM, Capitant C, Spire B, Pialoux G, Cotte L, Charreau I, et al. On-demand preexposure prophylaxis in men at high risk for HIV-1 infection. N Engl J Med. 2015;373(23):2237-46.

86. Patel P, Borkowf CB, Brooks JT, Lasry A, Lansky A, Mermin J. Estimating per-act HIV transmission risk: a systematic review. AIDS. 2014;28(10):1509-19.

87. Boily M-C, Baggaley RF, Wang L, Masse B, White RG, Hayes RJ, et al. Heterosexual risk of HIV-1 infection per sexual act: systematic review and meta-analysis of observational studies. Lancet Infect Dis. 2009;9(2):118-29.

88. Prabhu S, Harwell JI, Kumarasamy N. Advanced HIV: diagnosis, treatment, and prevention. The Lancet HIV. 2019;6(8):e540-e51.

89. Centers for Disease Control and Prevention. Updated guidelines for antiretroviral postexposure prophylaxis after sexual, injection drug use, or other nonoccupational exposure to HIV— United States, 2016. 2016.

**C. The Netherlands Model**

**Table S4. Key model parameters of oral PrEP and CAB-LA for HIV prevention in the Netherlands**

| **Model parameters** | | | **Estimate or range^a^/IQR** | **Reference** |
| --- | --- | --- | --- | --- |
| Duration of disease stages | | |  |  |
|  | Acute stage | | 10–16 weeks | [1] |
|  | CD4+ T-cell count 350–500 cells/µL | | 2.9–3.1 years | [2] |
|  | CD4+ T-cell count 200–349 cells/µL | | 3.6–3.9 years | [2] |
|  | CD4+ T-cell count < 200 cells/µL | | 13–25 months | [2] |
| Infectivity per partnership transmissibility per year | | |  |  |
|  | Acute stage | | 0.030–0·61 | [3]; Model calibration |
|  | Chronic stage | | 0.027–0·21 | [3]; Model calibration |
|  | AIDS stage | | 0.008–0·27 | [3]; Model calibration |
| Proportion MSM in sexual partnership groups | | |  |  |
|  | Highest | | 11% (11-12% IQR) | Model calibration, the sum of the three groups was equal to 100% |
|  | 2^nd^ highest | | 14% (12-16% IQR) |  |
|  | 3^rd^ highest | | 19% (14-23% IQR) |  |
|  | Lowest | | 56% (49-61% IQR) |  |
| Number partners/2 years in sexual partnership groups | | |  |  |
|  | | Highest | 43.88 (34.95-55.27 IQR) | Model calibration |
|  | | 2^nd^ highest | 9.78 (7.99-12.05 IQR) |  |
|  | | 3^rd^ highest | 2.65 (1.85-3.67 IQR) |  |
|  | | Lowest | 0.20 (0.14-0.29 IQR) |  |
| Mortality rates per year | | |  |  |
|  | Population | | 0.0155 | [4] |
|  | Chronic HIV stage | | 0.114 | [4] |
|  | AIDS stage | | 0.648 | [4] |
|  | On treatment | | 0.0184 | [4] |
| Oral PrEP regimens discontinuation rate | | | 0.62/year (0.48-0.79 IQR) | Model calibration |

Note: AIDS: acquired immunodeficiency syndrome; HIV: human immunodeficiency virus; IQR: interquartile range; PrEP: pre-exposure prophylaxis.

^a^ All ranges were uniformly distributed.

***References***

1. Pilcher CD, Joaki G, Hoffman IF, Martinson FE, Mapanje C, Stewart PW, et al. Amplified transmission of HIV-1: comparison of HIV-1 concentrations in semen and blood during acute and chronic infection. Aids. 2007;21(13):1723-30.

2. Lodi S, Phillips A, Touloumi G, Geskus R, Meyer L, Thiébaut R, et al. Time from human immunodeficiency virus seroconversion to reaching CD4+ cell count thresholds <200, <350, and <500 Cells/mm³: assessment of need following changes in treatment guidelines. Clin Infect Dis. 2011;53(8):817-25.

3. Sood N, Wagner Z, Jaycocks A, Drabo E, Vardavas R. Test-and-treat in Los Angeles: a mathematical model of the effects of test-and-treat for the population of men who have sex with men in Los Angeles County. Clin Infect Dis. 2013;56(12):1789-96.

4. Nakagawa F, Lodwick RK, Smith CJ, Smith R, Cambiano V, Lundgren JD, et al. Projected life expectancy of people with HIV according to timing of diagnosis. Aids. 2012;26(3):335-43.

5. van de Vijver D, Richter AK, Boucher CAB, Gunsenheimer-Bartmeyer B, Kollan C, Nichols BE, et al. Cost-effectiveness and budget effect of pre-exposure prophylaxis for HIV-1 prevention in Germany from 2018 to 2058. Euro Surveill. 2019;24(7).

6. RIVM. Aantal PrEP gebruikers per GGD binnen regeling PrEP: Overzicht per eind februari 2023. 2023.

7. van Sighem AI, Wit F, Boyd A, Smit C, Matser A, Reiss P. Monitoring Report 2022. Human Immunodeficiency Virus (HIV) Infection in the Netherlands. Amsterdam: Stichting HIV Monitoring, 2022. 2022 [Available from: https://www.hiv-monitoring.nl/en/resources/monitoring-reports.

**6.** **Model Comparison Modelling Assignment**

**JOINT PROJECT:**

**Assessing the impact and utility of long-acting PrEP to help control HIV epidemics in various risk populations and settings**

*Background:* Recently, two clinical studies (HPTN 083 and HPTN 084), have shown the superiority of long acting injectable PrEP (LA PrEP) over oral PrEP among MSM in North and South America, South Africa and India and women in Sub-Saharan Africa, respectively. The addition of this new prevention tool may be appealing to people at risk, who do not want or are unable to use or adhere to daily oral PrEP, which may reduce or widen inequity gaps when it becomes widely available. However, many issues need to be considered to determine how to assess and optimise the use of LA PrEP before it is approved by regulatory authorities, is manufactured in sufficient capacity, and becomes widely available to the public and rolled out more widely.

*Project objective*: The HPTN Modelling Centre and the HIV Modelling Consortium are collaborating to conduct a model comparison of the population-level impact of introducing LA PrEP, over different time horizons, derived from transmission-dynamic models of HIV in different geographic areas and selected risk populations.

Inclusion criteria: Suitable model criteria for inclusion in this modelling comparison exercise are i) transmission dynamic models of HIV transmission in heterosexual or MSM population; ii) stratified by risk level to define eligibility of PrEP use.

*Rationale:* The aims of this project are 1) to use existing models to provide and compare estimates of the population-level effectiveness and efficiency of expanding overall PrEP coverage by adding LA PrEP to the HIV prevention portfolio, and 2) conduct cost-effectiveness analysis to determine the threshold cost of LA PrEP which will likely make it cost-effective in different epidemic settings and risk populations. Ultimately, the results of this study will fill an important information gap for policy and provide the basis for recommendations for regional, national and local HIV policies as well as support strategic planning and allocation to key population programmes.

The main metrics to be assessed are as follows:

1. Population level effectiveness: relative reduction of cumulative new HIV acquisitions and HIV-related deaths over 10 and 20 years for each intervention scenario compared to baseline scenarios (status quo and expanded ART)

2. Efficiency: the number of HIV acquisitions prevented per person-year on LA PrEP for each intervention scenario compared to baseline scenarios over 10 and 20 years

3. Cost-effectiveness: The cost per acquisition and DALY averted over 10 and 20 years for each intervention scenario compared to baseline scenarios

**SUMMARY**

**Common assumptions**

- For comparability, we are requesting each modelling team use the same estimates of:
  - LA PrEP efficacy in reducing HIV acquisition risk
  - LA PrEP retention rate

Estimates based on data from HPTN 083 and HPTN 084 will be provided to the teams.

- All teams are expected to report results with 100 calibrated parameter sets which best represent (fit) the HIV epidemic in their setting of choosing. Same 100 parameter sets should be used to simulate both, base-case and intervention scenarios.
- All base-case and intervention scenarios should be simulated for 20 years forward, starting on Jan 1, 2022:

**Base-case scenarios**

All teams are expected to simulate a base-case scenario in which:

- rates of ART and viral suppression (VS) management in the model (including initiation and dropout rates) are kept at their current level for the next 20 years

– oral PrEP use is expanded according to local eligibility criteria, availability and acceptability

In future iterations using base-case scenarios assuming improvement in ART coverage and VS toward reaching goals 95-95-95 by 2030 may be requested.

**Intervention scenarios with added LA PrEP**

All teams are expected to simulate some or all of the intervention scenarios listed below:

| **Intervention components** | **Scenarios to be simulated** |
| --- | --- |
| Overall PrEP coverage (oral + LA PrEP coverage combined) | - 15%, 30%, 40% and 50% overall PrEP coverage achieved after 5 or 10 years |
| Percentage of oral PrEP users switching to LA PrEP | - 0%, 15%, 30% and 50% of current or projected oral PrEP users switch to LA PrEP |
| Distribution of additional LA PrEP | - based on current PrEP eligibility criteria  - proportional (across risk and age groups) |

Details on intervention scenarios:

1. Targeted PrEP coverage needs to be reached by Jan 1, 2027 (if 5 years target) or Jan 1, 2032 (if 10 years target) but not exceeded by more than 2 percentage points

2. Only scenarios with targeted PrEP coverages greater than the projected oral PrEP coverage in the base-case scenario should be simulated

3. The percentage of existing oral PrEP users who switch to LA PrEP are assumed to do so at the start of the simulations on Jan 1, 2022. Same percentage of future (projected) oral PrEP users are assumed to initiate LA PrEP instead of oral PrEP.

4. If targeted overall PrEP coverage implies additional LA PrEP usage, beyond projected oral PrEP usage in the base-case scenario, which is distributed based on PrEP eligibility AND eligible subgroups are large enough to achieve the overall PrEP coverage target then only people from eligible subgroups should initiate LA PrEP.

5. If targeted overall PrEP coverage implies additional LA PrEP usage, beyond projected oral PrEP usage in the base-case scenario, which is distributed based on PrEP eligibility AND eligible subgroups are not large enough to achieve the overall PrEP coverage target then different rates of initiating LA PrEP should be assumed for eligible and ineligible subgroups which ensure that i) overall PrEP coverage target is achieved and ii) 90% of the eligible subgroups use PrEP (oral or LA) over the targeted period (5 years or 10 years).

6. If targeted overall PrEP coverage implies additional LA PrEP usage, beyond projected oral PrEP usage in the base-case scenario, which is distributed proportionally then equal rates of initiating LA PrEP should be assumed for eligible and ineligible subgroups which ensure that overall PrEP coverage target is achieved over the targeted period (5 years or 10 years).

An example of a possible intervention scenario:

- Oral PrEP introduced in 2018
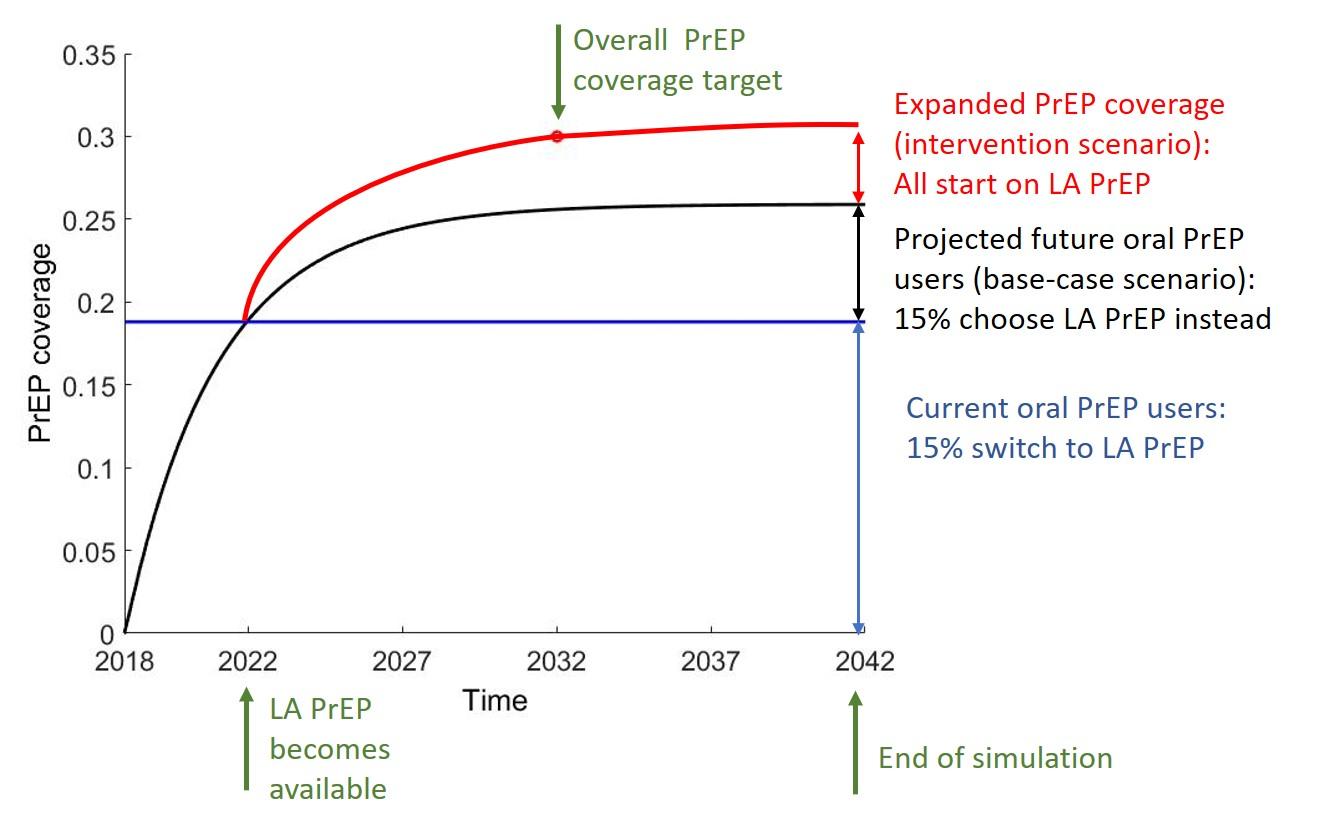

- 18.8% oral PrEP coverage in 2022
- 25.6% expected oral PrEP coverage in 2032
- 30% overall PrEP coverage target
- Achieved in 10 years (2032)
- 15% of current/potential oral PrEP users choose LA PrEP when available

Figure SEQ Figure \* ARABIC 1. Expected PrEP use over time for a specific intervention scenario

**Outcomes to be reported**

All teams are expected to report the following groups of outcomes for all 100 simulations per scenario:

- HIV care cascade over time
- Number of people eligible for PrEP over time
- Number of people using PrEP (oral and LA) over time
- Distribution of untreated HIV-positive people by CD4 count over time
- Annual number of HIV acquisitions (overall and among PrEP users)
- Annual number of HIV-related deaths (overall and by age groups)

More details on the expected outcomes and the exact format of the expected results can be found in the attached Excel spreadsheet (Scenario template)

**Additional information to be reported**

All teams are expected to provide additional details on their models to help interpreting results and ensure comparability. This includes information on:

- Model type
- Epidemic settings, simulated population and how it is stratified in the model
- Assumption about oral PrEP use (past, present, future)
- Other interventions included in the model
- Details on model calibration
- Handling of drug-resistance

Estimates of the following outcomes by population subgroup at the time of introducing LA PrEP (Jan 1, 2022) are expected:

- Population size
- HIV prevalence
- HIV incidence (the year before)
- Condom use
- oral PrEP coverage
- ART coverage
- Viral suppression

The additional data should be provided in the attached Excel spreadsheet (Model details)

**Submitting results**

Results for each scenario should be saved in separate .csv files. The base-case file should be named **sc_base.csv**. Each intervention scenario file should be named **sc_cX_aY_sZ_W.csv** where:

- X is the percentage (15, 30, 40, 50) overall PrEP coverage targeted;

- Y is the time in years (5,10) when this coverage must be achieved;

- Z is the percentage (0, 15, 30, 50) of current/projected oral PrEP who switch to LA PrEP and

- W indicates if additional LA PrEP is distributed among PrEP eligible individuals only (W=e) or proportionally among all uninfected individuals (W=p)

Example: **sc_c30_a10_s15_e.csv** should be the file holding outcomes of the scenario illustrated in Figure 1 assuming that 30% overall PrEP coverage is achieved over 10 years with 15% of the existing/projected oral PrEP users switching to LA PrEP and additional LA PrEP being distributed among PrEP eligible individuals only.
